# Towards the Internet of Smart Trains: A Review on Industrial IoT-Connected Railways

**DOI:** 10.3390/s17061457

**Published:** 2017-06-21

**Authors:** Paula Fraga-Lamas, Tiago M. Fernández-Caramés, Luis Castedo

**Affiliations:** Department of Computer Engineering, Faculty of Computer Science, Universidade da Coruña,15071 A Coruña, Spain; tiago.fernandez@udc.es (T.M.F.-C.); luis.castedo@udc.es (L.C.)

**Keywords:** IoT, IIoT, internet of trains, railway safety, rail planning and scheduling, predictive maintenance, WSN, railway enhanced services, freight transportation, cyber security

## Abstract

Nowadays, the railway industry is in a position where it is able to exploit the opportunities created by the IIoT (Industrial Internet of Things) and enabling communication technologies under the paradigm of Internet of Trains. This review details the evolution of communication technologies since the deployment of GSM-R, describing the main alternatives and how railway requirements, specifications and recommendations have evolved over time. The advantages of the latest generation of broadband communication systems (e.g., LTE, 5G, IEEE 802.11ad) and the emergence of Wireless Sensor Networks (WSNs) for the railway environment are also explained together with the strategic roadmap to ensure a smooth migration from GSM-R. Furthermore, this survey focuses on providing a holistic approach, identifying scenarios and architectures where railways could leverage better commercial IIoT capabilities. After reviewing the main industrial developments, short and medium-term IIoT-enabled services for smart railways are evaluated. Then, it is analyzed the latest research on predictive maintenance, smart infrastructure, advanced monitoring of assets, video surveillance systems, railway operations, Passenger and Freight Information Systems (PIS/FIS), train control systems, safety assurance, signaling systems, cyber security and energy efficiency. Overall, it can be stated that the aim of this article is to provide a detailed examination of the state-of-the-art of different technologies and services that will revolutionize the railway industry and will allow for confronting today challenges.

## 1. Introduction

The future of the railway industry is expected to rely upon smart transportation systems that leverage technologies over a large rail network infrastructure to reduce its life-cycle cost. New services, such as integrated security, asset management, and predictive maintenance, are expected to improve timely decision-making for issues like safety, scheduling, and system capacity. Smart railways represent a combination of interconnected technological solutions and components, as well as modern transportation infrastructure like automatic ticketing systems, digital displays, and smart meters. Likewise, these systems require seamless high data rate wireless connectivity and integrated software solutions to optimize the usage of assets, from tracks to trains, to meet the ever-growing demand for energy-efficient and safer services. The driving factors of the smart railways are expected to enforce the growth of the industry. These factors include the increasing importance of sustainability, government regulations, demographics (i.e., growing traffic of passengers and freight, aging population, and rapid urbanization), macroeconomics (i.e., limited public funding and government deficit, government initiatives and partnership models), microeconomics (i.e., price sensitivity, demands for an improved passenger experience, stakeholders interests), the growing importance of smart cities, the incredible pace of telecommunications and technological change, and the need for mobility.

The global smart railway market is estimated to grow from USD 10.50 bn last year to USD 20.58 bn by 2021, at a Compound Annual Growth Rate (CAGR) of 14.4% [1]. Moreover, according to the International Transport Forum of the Organisation for Economic Co-operation and Development (OECD), by 2050, passenger mobility will increase by 200–300% and freight activity by as much as 150–250% with respect to 2010 [2]. It is expected that these figures impact on each and every component of the value chain of the industry, from passenger service to the back-end organization.

In addition, the complexity of high-speed railway networks has been previously studied by different research initiatives, which were primarily aimed at fostering transportation quality. Among their diverse strategic goals is the introduction of advanced communication technologies, which allows for providing improved services and for coping with the rapidly changing needs of the market [3].

Current European railway communications technology was built in the beginning of the 90 s taking into consideration well-established standards with potential to deliver the railway services at that time [4].

The inception of smart railways began with the evolution of Global System for Mobile Communications-Railways (GSM-R), which is considered to be the keystone of rail industry transformation. Rail operators mainly use GSM-R for operational voice and data communications. Over a period of time, innovation in wireless communications technologies offered reliable transmission of video and data services for long distances. In the 2000s, the introduction of novel technological solutions and various digital devices projected new application areas, such as the provision of information about the rails to passengers, the Communication-Based Trail Control (CBTC), rail traffic management systems, and Positive Train Control (PTC) solutions. However, the rail industry underwent a major revolution after 2005 with the appearance of Internet of Things (IoT) and the adoption of smart city projects, which led to the development of solutions like smart ticketing, passenger infotainment, rail analytics, and dynamic route scheduling and planning. Industrial IoT-based solutions have eventually reinforced competitive advantages and have also uncovered new business models that are already impacting the global rail industry.

However, factors such as operational inefficiency, the lack of infrastructure and interoperability, high initial cost of deployment, and the integration complexities over legacy systems and the network, may hinder the rail industry growth. Moreover, legacy infrastructure, aging communications systems, and the slow adoption of automation and protective technology in this scenario pose enormous safety risks. Related to the issues of safety and connectivity is security. As rail systems rely more and more on wireless connectivity, they become more vulnerable to outside interference, intrusion and cyber attacks. The consequences of even a small disruption become particularly severe as trains become more powerful, carry more passengers, and travel faster. Systems that are mission-critical for safe operation can be compromised by a simple electronic device or a small piece of malicious code. When passenger safety and lives are at stake, strong security becomes a fundamental requirement. Nowadays, the main challenges when enhancing rail transport can be summarized as [5]:

- Increase efficiency and competitiveness: railways face ferocious competition from other modes (for example, the road sector provides attractive, cost-effective, reliable, flexible, and convenient door-to-door transport of freight and passengers across borders). In Europe, the challenge is further increased by a fragmented rail market, with numerous national systems for rail signaling and speed control. Thus, interoperability represents a key challenge for the free flow of rail traffic.
- Reduce rail noise and vibration, particularly in urban areas.
- Reduce greenhouse gas emissions. Although rail transport compares favorably to other transport means in terms of environmental impact, it can be further improved.
- Safety and security [6]: rail safety in the European Union (EU) is among the highest in the world. Rail incidents (accidents, terrorism...) are not frequent and cause a relatively low toll of deaths, but often involve a substantial number of people. In order to maintain and enhance security, interoperable and harmonized safety standards are required.
- Reduce operation and maintenance costs, augment the capacity of the rail network.

As it will be explained in the next section, considering the diversity of scenarios, the network architecture should include different types of access networks and technologies at different frequency bands in order to fulfill different operational requirements.

This review introduces a comprehensive analysis of the evolution of the communications in the European railways since the deployment of GSM-R. It examines the different alternatives proposed over time and how railway requirements, specifications and recommendations have evolved in recent years. Unlike recent literature, the main contribution of this work focuses on presenting a holistic approach to IIoT applied to railways with a thorough study of the most relevant technologies (like the communications network). Its aim is to envision the potential contribution of enabling technologies for revolutionizing the industry and confront today challenges.

**Figure 1:**
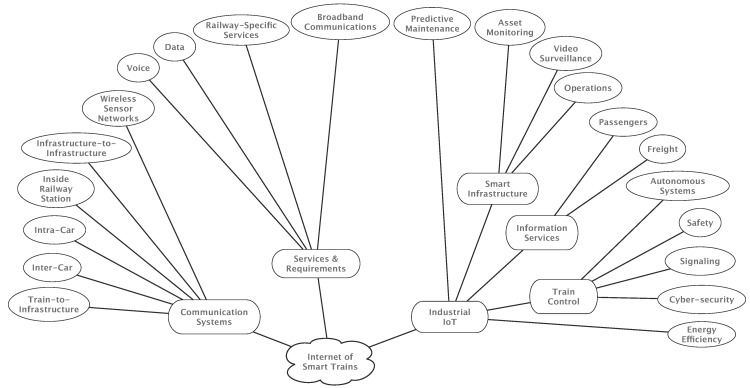
Overview of the topics related to the Internet of Smart Trains that are covered in this article.

The rest of this article is organized as follows. Section 2 provides a brief introduction of the main railway scenarios, examining the communications technologies and architectures used nowadays. Section 3 reviews the basic railway-specific requirements and services offered by GSM-R. Section 4 analyzes the factors that influence the deployment of LTE, and what is necessary to comply with the specific requirements of railway services. The advantages of the newest generation of communications systems for the railway environment are also explained together with the roadmap to ensure a satisfactory migration from GSM-R to LTE-R. Section 5 describes the rise of industrial IoT and the paradigm of Internet of Trains. Furthermore, the main industrial developments are described. Section 6 reviews the main short and medium-term IIoT-enabled services for smart railways. Finally, Section 7 is devoted to the conclusions. For the sake of clarity, Figure 1 shows an overview of the contents covered by the survey.

## 2. Communication Systems in Railway Scenarios

**Figure 2:**
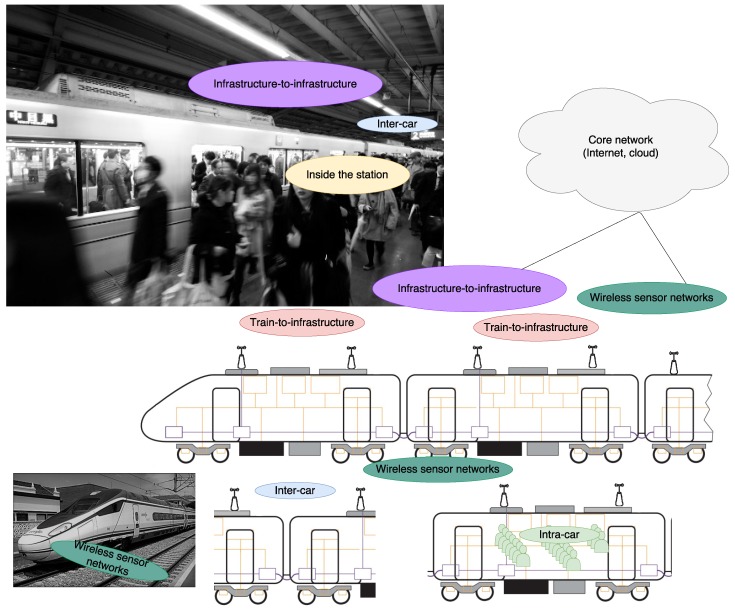
Railway communications scenarios (Renfe AVE train and train station pictures are under Creative Commons License). Color meaning: pink (train-to-infrastructure communications), blue (inter-car communications), light-green (intra-car communications), yellow (communications inside the station), purple (infrastructure-to-infrastructure communications), and dark green (wireless sensor networks).

Railway lines can be categorized mainly into one of four classes: urban, urban/inter-city, inter-city and/or high-speed. It is necessary to analyze lines or networks separately, given that their differences may have impact on their requirements (Table 1). Furthermore, railway communication systems can be divided into three main application groups: safety and control, operator, and customer oriented networks. In this Section, the communications in the most representative railway scenarios (Figure 2) are described: train-to-infrastructure communications, inter-car communications, intra-car communications, communications inside the station, infrastructure-to-infrastructure communications, and wireless sensor networks. In the following subsections, future directions of wireless systems in railways are addressed.

### 2.1. Train-to-Infrastructure Connection

This scenario requires two types of links among the Access Point (AP) transceivers located in the train and the fixed network infrastructure. These links must be bidirectional, with high data rates and latencies lower than 100 ms while traveling at speeds up to 350 km/h or even higher [3]. Jointly with an availability of 98–99% mandatory to comply with Reliability, Availability, Maintainability and Safety (RAMS) requirements.

Several works exist in the literature related to the characterization of train-to-ground wireless links [7]. Generally, train-to-infrastructure systems communicate with wayside units using GSM-R or IEEE 802.11. For instance, the effect that structures like viaducts, bridges and terrain cuttings can cause in GSM-R has been analyzed exhaustively in the literature [8]. Furthermore, in the case of high-speed scenarios, Wang et al. [9] present a survey on channel measurements and models.

### 2.2. Inter-Car Connection

Wireless communications and optical fiber can both be employed for inter-car communications. Nevertheless, the latter is less advised since it may be costly to wire a train for network access, and rewiring may be necessary each time the train is reconfigured [3].

This scenario demands high data rates and low latencies. The APs are rearranged in each wagon such that every one acts as a client station for the AP in the previous car, and as an AP for all the stations within its car. The propagation channel of these communications has been investigated in literature. An example of wireless channel measurement in order to characterize the propagation environment for inter-car communications is described in [10]. Moreover, a measurement and analysis of a channel considering the use of Trans European Trunked RAdio (TETRA) is presented in [11].

The communications between vehicles cover several use cases. For example, the information on-the-fly between two vehicles. It is frequently a disabled one, out of range of a communication network that transmits information to another vehicle passing nearby [12,13,14]. A different example to accelerate the coupling process is the virtual coupling of two vehicles (including car trains or wagons, subways and trams). Moreover, specific mechanical connectors that deteriorate rapidly under the rough vibration conditions in railway operations could be avoided. Nevertheless, for virtual coupling, train-to-train communications are essential to interconnect high-speed networks embedded in both vehicles.

Currently, the main technologies for inter-car communications are Wireless Fidelity (Wi-Fi), Dedicated Short-Range Communications (DSRC), and Worldwide Interoperability for Microwave Access (WiMAX). Another candidate technology for the wireless connection is Ultra-wideband (UWB), the IEEE 802.15.4a standard. The UWB links are more robust to frequency selective fading. IEEE 802.11p [15] may be an option if high data rates are not required. New technologies at 60 GHz carrier frequencies like mmWave, IEEE 802.11ad and Machine-to-Machine (M2M) communication systems are also being considered.

### 2.3. Intra-Car Communication Networks

Since the early 1980s, on-board communication networks were installed on trains to reduce the wiring used to transfer information between distinct devices like Human-Machine Interface (HMI) or Heating, Ventilation and Air Conditioning (HVAC). Multiplexing digital information techniques over a serial cable have tried to replace most of the classical point-to-point copper lines or so-called train lines. In 1999, wired communication networks were standardized for on-board railway applications (the standard was superseded in 2010 [16]) by defining Wire Train Bus (WTB) and Multifunction Vehicle Bus (MVB) networks for Train Control and Management System (TCMS) application.

Standards like CANOpen, LonWorks, Profibus, WorldFIP, Leaky Coaxial Cable (LCX) or Train Communication Network (TCN) are deployed either for metro or trains. Since the 2000s, manufacturers considered the Real-Time Ethernet (RTE) technologies by adding new standards to IEC 61375 standard series [17], such as Ethernet Train Backbone (ETB) or Ethernet Consist Network (ECN). Besides the control-command functions provided by classical field bus technologies, RTE provides Internet Protocol (IP) traffic. For example, a wired Ethernet network could be taken in consideration, but it implies high installation costs. In recent years, Power Line Communication (PLC) technology has experienced significant developments. A review on railway embedded network solutions is presented in [18].

In this scenario the links created by the APs provide wireless access to the passengers and to the sensors inside the car. Such a scenario is prone to backscattering, which results in attenuations [13]. Three wireless access modes can be enabled to provide good coverage inside the cars:

- Direct transmission from the Base Station (BS). The problem in this mode is that the signal from the BS has to penetrate into the car, what derives in a loss of up to 24 dB that needs to be compensated by incrementing the transmission power and the receiver sensitivity.
- Use of in-car repeaters. The signals from the BS are received by an on-vehicle transceiver, which forwards them to a micro-base or to a Wi-Fi signal repeater. Note that this scheme increases the signal power through repeaters, but these additional devices increase the communications delay significantly. For this reason, a topic under research is the design and implementation of transmission schemes that offer good coverage for repeaters at high speeds.
- Two-hop access mode. In this mode the transmission requires first to travel from the BS to the antennas located on top of the train, and then to the receiver placed inside the train. This approach usually avoids the penetration losses related to a direct transmission from the BS. Nevertheless, it is worth noting that, since high frequency bands have large attenuations and path losses, its use may derive in a limited coverage.

When the second and third access modes are used, it is necessary that the signal penetrates the vehicle, what usually causes interferences. Furthermore, the selection of a proper communication technology depends on the bandwidth requirements, which are mainly conditioned by the services provided and by the number of simultaneous users. Assuming around 130–180 passengers per car, a bandwidth of up to 3.6 GHz would be needed if half of the passengers demand real-time HD video. If the streaming service has to provide bidirectional HD video (i.e., video conference), the bandwidth requirement may double. These bandwidth requirements cannot be fulfilled by LTE, which only makes use of 20 MHz instead of the 7.2 GHz that would be demanded. The solution might be provided by mmWave/sub-mmWave bands at 28 and 300 GHz, and the 5G communication systems, which offer larger bandwidths and higher data rates. For instance, massive Multiple-Input Multiple-Output (MIMO) and 3D beamforming may be used with many users to enhance the system capacity [3]. For wide area coverage, signals at frequency bands below 6 GHz are needed.

#### Main Technologies for Intra-Car Communication Networks

Several technologies are embedded in the Train Access Terminal (TAT) to provide a continuous connection. Furthermore, they are able to link the train to the Internet backbone and to provide Internet on-board. Apart from the bandwidth requirement, among the criteria to select a specific technology are generally the connection quality (i.e., the signal strength), delay, throughput, security, and cost. Two major families of technologies may be considered [19,20]:

- Satellite solutions. Distinct types are available (i.e., Geostationary Orbit (GEO), Medium Earth Orbit (MEO), Low Earth Orbit (LEO)) with different frequency bands and that may provide unidirectional or bidirectional communications. Satellites are used for both locating trains (aided by Global Navigation Satellite Systems (GNSS) systems [21], like GPS, the European GALILEO, the Russian GLONASS or the Chinese BEIDOU) and communicating with the wayside equipment.
- Terrestrial solutions. They can be grouped into two main categories: (a) technologies that rely on existing networks (i.e., public cellular networks), and (b) technologies that require ground infrastructure to be deployed: leaky coaxial cable, Wi-Fi, WiMAX, radio-over-Fiber, and optical solutions.

Apart from legacy systems (usually analog), the trend of applying wireless systems in railways is still in its first decade of life. There are three types of systems. First, those based on open standards: TETRA, General Packet Radio Service (GPRS), and IEEE 802.11 family of standards; second, open standards with slight modifications on some layers (e.g., GSM-R); finally, proprietary wireless communication solutions have also its niche in the market. Traincom by Telefunken [22] or FLASH-OFDM [23] are good examples with a great acceptance in the railway sector.

Nowadays, GSM-R is the most widely used communications system between trains and the different elements involved in operation and control within the railway infrastructure. It operates in 38 countries across the world, including all member states of the European Union (EU) and countries in Asia, America, and northern Africa [24]. Two frequency bands were reserved by the European Telecommunications Standards Institute (ETSI) for railway communications in Europe in 1995, which are 921–925 MHz for Downlink (DL) and 876–880 MHz for Uplink (UL). For each band, it is possible to allocate 19 subcarriers of 200 kHz, including a guard band. Each subcarrier supports 8 data or voice channels.

A Wireless Local Area Network (WLAN) technology such as Wi-Fi represents the most common deployment on-board, and it is accepted that the replication of Wi-Fi APs within the train is the best approach to connect trains with a client interface. The delivery of broadband Internet access to trains has been previously analyzed in the literature and some authors have presented surveys that compare different technologies for such a purpose (e.g., IEEE 802.11, TETRA, satellite) [25].

Due to the rapid changes in technology, it is clear that railways will have to evolve to keep up with their pace. With such an aim in mind, in recent years operators have included in their systems different emerging technologies. For example, WiMAX was tested for train-to-ground deployments in order to provide Internet services to the passengers [26]. Other WLAN-based networks have been evaluated to deliver train operation traffic but, until the development of the IEEE 802.11ac standard, there was a lack of essential Quality of Service (QoS) features related to traffic policy enforcement, end-to-end resource management or traffic admission.

Likewise, new technologies like Wireless Gigabit (WiGig) or Light-Fidelity (Li-Fi) will have to be considered in the medium-term [20]. On the one hand, WiGig (IEEE 802.11ad), promoted by the Wi-Fi Alliance, operates at the unlicensed 60 GHz band (in Europe 9 GHz of bandwidth from 57 to 66 GHz). It offers high-speed, low latency, a throughput of up to 7 Gbps with a transmission distance of up to ten meters, and protected connectivity between nearby devices. Its Medium Access Control (MAC) layer is extended and it provides backward compatibility with the IEEE 802.11 standard. When operating in the mmWave domain, beamforming techniques are needed to overcome the path loss from transmitter to receiver, what was not an issue for IEEE 802.11 a/b/g/n due to the use of omnidirectional antennas. On the other hand, Li-Fi (IEEE 802.15) is a 5G Visible Light Communication (VLC) system that makes use of light form diodes to deliver mobile and high-speed communications. For instance, Li-Fi uses amplitude modulation of light sources in accordance to an standardized protocol. Its main drawbacks are that communications require to switch on a light during transmissions and that mobility is not possible. For example, the France’s national state-owned company *Société Nationale des Chemins de Fer* (SNCF) has been interested in Li-Fi during the last years. For instance, recent applications involving mass-market devices only have DL communications implemented. A project between Lucioum Company and Leti have developed a bidirectional Li-Fi modem that allows for providing wireless Internet access of up to 20 Mbps [27]. Furthermore, Oledcomm provides Internet access via Li-Fi [28]. On-board Internet transmitting via individual lights of the passengers is a topic under research.

IIoT can harness the surplus capacity offered by mobile operators in order to provide novel services. In this way, 4G and 5G broadband can help smart railways to attract users from other competing transport means thanks to their coverage and the possibility of offering services like live-video streaming or mobile ticketing. Moreover, safety in railways can be improved through driver advisory systems (i.e., on-board Closed-Circuit Television (CCTV) recordings transferred to a Train Control Center (TCC)), train diagnostics, and driver vigilance detection (for instance, the driver’s health can be monitored by using a wireless wearable EEG [29]).

Nevertheless, quite a few companies have established a quota limit on throughputs. For example, Amtrak (Washington, DC, USA) blocks the access to streaming media and limits file downloads to 10 MB [30]. Such a quota limit is also employed in the NS Dutch railways, which provide a speed of 150 kbps per user. Most developments were first rolled out in the 2000s, and they have been upgraded with the first deployments of 4G technologies and the usage of the Ka band for satellite communications. Nevertheless, although 5G systems are currently discussed in 3GPP, commercial devices will not be available until 2020. A recent document that sets out requirements and guidance for Internet provision is presented in [31].

### 2.4. Inside the Railway Station

The railway station is a scenario characterized by a semi-closed scene with a crowd of people. The links created by the APs provide wireless connectivity to the users, who are usually interested in broadband communication services. For such a purpose, a fixed/wireless communication infrastructure has to be deployed in the stations, which might support operational (e.g., fire protection, automatic doors, surveillance) and commercial services (e.g., cash desks).

For instance, massive MIMO technology is an appropriate choice for providing communications in railway stations and inside cars, since it is able to achieve high spectral efficiency, high data rates, and high energy efficiency. Moreover, the transmission modes can be adapted dynamically to the presence of multiple simultaneous users by grouping hundreds of specifically designed antennas.

### 2.5. Infrastructure-to-Infrastructure

Infrastructures are connected in real-time and require bidirectional links with high data rates and low latencies. The information is transferred between the cameras or the IoT infrastructure and the APs deployed on the trains, stations, platforms, and the wayside along rail tracks.

Table 2 reviews the main characteristics of the technologies that are commonly used in the scenarios previously described.

### 2.6. Wireless Sensor Networks

As a result of the combination of the latest advances in electronics, networking, and robotics, it is feasible to develop advanced sensor systems for different sectors and applications: energy efficiency [32], Industry 4.0 [33,34], home automation [35], public safety and defense [36,37], precision agriculture [38] or transportation [39]. Furthermore, Wireless Sensor Networks (WSNs) have evolved into an integral part of the protection of mission-critical infrastructures [36]. Today, WSNs are used in the scenarios previously described, where sensors can be on top of the train, inside, beside, interacting between railway vehicles and tracks, or even as part of the station infrastructure. An example in the railway station could be the ticket validation equipment based on Low Frequency (LF), 125–135 kHz, and High Frequency (HF), 13.56 MHz RFID bands, or vehicle tagging based on Ultra High Frequency (UHF) RFID solutions [40] in the 865–869 MHz band.

The sensors make use of the protocols listed in Table 3 to communicate and organize themselves. The information collected is transmitted to APs that utilize more powerful communication technologies, such as the ones cited in the previous sections (e.g., GPRS, Wi-Fi, WiMAX, LTE), to transmit the acquired data to TCCs. In Section 6.2, the applications of WSNs will be further explained.

## 3. Overview on the Railway Applications Offered by GSM-R

This section details the steps related to the adoption of GSM-R and reviews the main railway-specific services and requirements.

### 3.1. GSM-R: The Solution Preferred

The *Union Internationale des Chemins de Fer* (UIC) selected the GSM technology after comparing it with TETRA in terms of usability in railway scenarios. Moreover, GSM was supported by the GSM Association (GSMA) and it was standardized by ETSI as GSM Release 99. After a thorough analysis, GSM-R was eventually standardized by the European Railways and the UIC. In Europe, a relevant initiative for the evolution of the communications was the European Integrated Railway Radio Enhanced NEtwork (EIRENE) project. This cooperation was participated by the European Commission (EC), ETSI and several railway operators. EIRENE was aimed at specifying the requirements for railway mobile networks. To reach such a goal, a functional group and a project team were established within the project. The functional group was responsible for defining the Functional Requirements Specification (FRS), which guaranteed the interoperability across borders. Regarding the project team, it was focused on defining the System Requirements Specification (SRS). The SRS details the technical features related to operations, which involved the identification and specification of supplementary Advanced Speech Call Items (ASCI) features [41].

In 1995, a first draft of the EIRENE specifications was released. At the same time, the UIC became involved in the Mobile Radio for Railway Networks in Europe (MORANE) project, which also included the participation of the EC, the major railways of Italy, France and Germany, and a number of GSM suppliers. The objective of MORANE was to design and build prototypes of a new radio system that met the functional specifications and the system requirements proposed. Railways from all over Europe signed the Memorandum of Understanding (MoU) in 1998 and, in 2009, more companies were added, including railways outside Europe. In 2000, seventeen railway companies signed an Agreement on Implementation (AoI) to deploy national GSM-R networks no later than 2003. Thereafter, GSM-R became the reference communication technology in railways until today, when the evolution of the demand and the emergence of new technologies are fostering the research on alternative solutions.

It is publicly recognized that GSM-R is not well-suited for services such as automatic pilot applications or for provisioning on-board Internet to the train staff and passengers [42]. GSM-R (based on GSM Phase 2 and Phase 2+ recommendations) was designed aiming to provide the maximum redundancy while achieving the maximum system availability. GSM-R provision two fundamental services: voice communications and the transmission of European Train Control System (ETCS) messages.

The definition of European Rail Traffic Management System (ERTMS) was the result of the European efforts to promote interoperability. ERTMS includes three levels. Among them, ERTMS levels 2 and 3 employ GSM-R as the basis that supports communications. In Europe, a 4 MHz bandwidth is reserved for such communications. The main elements of ERTMS are:

- ETCS: it allows for automating train control. It consists of a Radio Block Center (RBC) and a Lineside Electronic Unit (LEU). ETCS can be divided into three levels:
  − ETCS level 1: the location of the train is determined by traditional means (i.e., no beacons are used for locating the train), whereas communications between fixed safety infrastructure and trains are performed by means of beacons (transponders placed between the rails of a railway track). GSM-R is only used for voice communications.
  − ETCS level 2: the communications between trains and the railway infrastructure are continuous and supported by GSM-R technology. The location of the train is estimated by means of fixed beacons.
  − ETCS level 3: the integrity of the train elements is checked at the train, thus no devices are required in the track. Fixed beacons are used to locate the train.
- EURORADIO GSM-R: radio infrastructure.
- EUROBALISE: beacons allowing for locating the trains accurately.
- EUROCAB: on-board management system that includes European Vital Computer (EVC), Driver-Machine Interface (DMI), and measurement devices such as odometers.

The ERTMS/GSM-R project was initiated by the UIC to unite existing and future developers to upgrade the GSM-R specifications. The collaboration continues today as an alliance between ETSI and the GSM-R industry. FRS version 8.0.0 [43] and SRS version 16.0.0 [44] (European Railway Agency (ERA) GSM-R Baseline 1 Release 0) were published in December 2015, representing the latest specifications. The mentioned documents describe the Mandatory (M) requirements regarding the interoperability of railways, according to Directive 2008/57/EC [45], and the requirements towards an IP-based core network architecture [46].

### 3.2. Railway-Specific Services and Requirements

Following the last EIRENE specifications, the integrated wireless network should comply with the general and functional requirements under these four categories: Mandatory for Interoperability (MI), Mandatory for the System (M), Optional (O) or Not Applicable (NA), depending on the type of radio. Specifically, the following system services are required [47]:

- Services: voice, data, and call related features (Table 4).
- Voice Group Call Service (VGCS) conducts group calls between trains or Base Stations (BSs), or between station staff and trackside workers.
- Voice Broadcast Service (VBS) is used to broadcast recorded messages or announce operations to certain groups of trains or BSs. The call set-up required times are shown in Table 5, it shall be achieved in 95% of cases (MI). Furthermore, call set-up times for 99% of cases shall not be more than 1.5 times the required call setup time (MI).
- Functional addressing (FA): a train can be addressed by a number identifying its function.
- Location dependent addressing (LDA): calls from a train can be addressed based on its location.
- Shunting mode for communicating to a group involved in shunting operations.
- Railway specific features [43,48] include the set-up of urgent or frequent calls through single keystroke or similar; display of functional identity of calling/called party; fast and guaranteed call set-up; seamless communication support for train speeds up to 500 km/h; automatic and manual test modes with fault indications; control over mobile network selection; and control over system configuration.

EIRENE-compliant mobile devices must guarantee the core requirements specified in SRS, together with network requirements and configuration. Furthermore, in the case of high-speed railways [49], as it is presented in Table 1, speeds of at least 220 km/h shall be managed while enabling speeds over 280 km/h under some circumstances. In general, speeds of 200–220 km/h represent the threshold for upgraded conventional lines. Nonetheless, connectivity has to be guaranteed at a moving speed of 500 km/h, or even more [50].

QoS mechanisms have to ensure the enhanced Multi-Level Precedence and Pre-emption (eMLPP). Although current networks manage different QoS policies according to the traffic types, QoS for real-time applications shall be checked. QoS control is needed for resource management. Besides, as it will be further explained in the next Sections, strict latency requirements are needed for seamless operation (i.e., train status and location), and the Movement Authority (MA) permission between the in-service train and the control center (i.e., the connection establishment error ratio over one train line should be less than 1% per hour and 99% of ETCS data should have a maximum latency of <0.5 s [51,52]). Table 6 shows a summary of the main GSM-R QoS parameters jointly with their availability.

## 4. Long Term Evolution (LTE): One Step Ahead of Broadband Communication Systems

As a narrowband system, the main GSM-R shortcoming relates to its limited provision of advanced data services due to its lack of packet-switched transmissions (Table 7). For instance, in order to deliver burst low-rate ETCS data, connections need to take network resources continually even though not being used. The maximum transmission rate of GSM-R per connection is 9.6 kbps and the packet delay is in the range of 400 ms, which is sufficient only for applications with low demands [42].

Possible solutions to enhance its limited capacity (i.e., ETCS in high traffic areas) include an LTE micro-cell deployment or the usage of the ER-GSM band (includes standard and extended GSM 900 band) and changing to ETCS over packet-switched data using GPRS, Enhanced General Packet Radio Service (EGPRS) or Enhanced GPRS Phase 2 (EGPRS2) [53].

Despite the commitment of the GSM-R Industry [54] to support GSM-R until 2030, these shortcomings are encouraging the replacement for different system architectures mainly LTE/LTE-A. This will be performed by introducing a framework for Control, Command and Signaling Technical Specifications for Interoperability (CCS TSI) that will enable the migration of technologies that can be used by the trackside and on-board systems from GSM-R to a next-generation system.

In 2011, the LTE-Advanced (LTE-A) specification (Release 10) was introduced. LTE-A meets formally the requirements of International Telecommunication Union-Telecommunication Standardization (ITU-T) 4G technology definition known as IMT-Advanced, and the needs set by the operator-led alliance Next Generation Mobile Networks (NGMN). Release 10 provided a substantial uplift to the capacity and throughput and also took steps to improve the system performance for mobile devices located at some distance from a BS. Main features included: up to 3 Gbit/s (DL) and 1.5 Gbit/s (UL); carrier aggregation (CA), allowing for the combination of up to five separate carriers (20 MHz) to enable bandwidths up to 100 MHz, higher order MIMO antenna configurations, relay nodes to support heterogeneous networks deployments and enhanced Inter-cell Interference Coordination (eICIC). Release 11, functional freeze date including stable protocols in early 2013, included refinements to existing capabilities: enhancements to Carrier Aggregation, MIMO, relay nodes and eICIC, introduction of new frequency bands, and coordinated multi-point transmission/reception to enable simultaneous communication with multiple cells. Release 12 (functional freeze date in March 2015) introduces novel procedures for supporting diverse traffic types, a number of features to improve the support of HetNets, enhanced small cells for LTE, inter-site carrier aggregation, new MIMO and beamforming techniques and advanced receivers to maximize the potential of large cells Proximity Services (ProSe), MBMS enhancements, M2M applications, Self-organizing Networks and interworking between HSPA, Wi-Fi and LTE.

### 4.1. Current Status of Standardization

The UIC General Assembly of 2008 proclaimed that the rise of the LTE communication system was threatening the life-cycle of GSM technology and affecting the maintenance of the equipment. As a consequence, the UIC presented a technical report with the results after examining whether LTE communication system would be applicable to the integrated railway wireless network. The main outcomes were that LTE technology might be relatively suitable for the near-future railway communication network and meet various requirements of railway, but noted that additional research would be required.

Consequently, UIC officially launched the Future Railway Mobile Telecommunication System (FRMTS) project in 2013, which was aimed at developing the next-generation of railway communication solutions. In particular, UIC further strengthened its cooperation with the 3GPP standard body in order to reflect requirements of the next-generation network in LTE standards. Furthermore, the future of transportation will rely on intermodal networks combining the railway, the subway, and road transportation. For example, in this scenario train-to-ground communications systems will be based on Wi-Fi and LTE-A systems. In a EU context, with the aim of develop cross-border connections between neighboring countries, and foster innovation and competitiveness, the strategic long-term policy includes the completion of the single European Railway Area (SERA, Directive 2012/34/EU). The ERA (Regulation (EC) No 881/2004) was established to promote SERA and to help revitalize the sector while reinforcing its essential advantages in terms of safety. As from 2016, the ERA will unify the large number of national technical rules and develop an improved safety culture (common methods, targets and indicators) under the Directive (EU) 2016/798. After a three year transition period, the ERA will be empowered to issue single EU-wide certificates for rolling stock and railway undertakings.

Future wireless systems for railways need to address many issues like cost, spectrum allocation, and interoperability. Depending on the point of view, current technology is very expensive and sometimes it is not interoperable. GSM-R is, in some cases, a possible exception in terms of interoperability. However, open standards like 3GPP LTE imply heavy costs and a possible dependence on mobile operators, which is unlikely to be accepted by railway operators, apart from other disadvantages. Despite all these issues, one of the aims of several research groups in Asia and Europe, and projects all over the world (for example, the Roll2Rail Project [55]) is to study feasible wireless communication technologies for both train-to-infrastructure, inter-train and inside-train communications. 3GPP LTE introduced some functionality on its latest releases that targets the railway sector, like mobile relays, or Device-to-Device (D2D) communications. The maturity of LTE standards to address railway requirements is briefly summarized in Table 8. Moreover, it is also important to notice the ongoing work on Cognitive Radio (CR) [56]. The concept of CR has been highlighted as an attractive solution to the problem of the congestion of the radio spectrum occupied by licensed users. Furthermore, it is able to integrate all the heterogeneous wireless networks deployed.

Besides, LTE will be the baseline technology for the next generation of broadband public safety networks. National Public Safety Telecommunications Council (NPSTC), TETRA + Critical Communications Association (TCCA), and Critical Communication Broadband Group (CCBG) are contributing to the standardization processes [57]. This functionality will become available in products from 2017 onwards in LTE Release 13 (functional freeze date 2016). Release 13, in addition to enhancements to existing services and features, includes the completion of the first set of specifications covering mission-critical services, in particular Mission Critical Push To Talk over LTE (MCPTT). 3GPP continued to work on the characterization of carrier aggregation across additional band combinations to provide increased bandwidth within the limited frequency allocations to individual operators. Radio propagation was further improved with studies on MIMO antennas and sophisticated beamforming techniques. Other major advances achieved included enhancements to machine-type communications, public safety features, small cell dual-connectivity and architecture, indoor positioning, single cell point-to-multipoint and work on latency reduction.

In 2015, 3GPP began to work on the next generation cellular technology or 5G, with the aim of submitting a candidate technology to the IMT-2020 process. Meanwhile, work started on Release 14 and numerous features and studies had been defined including Multimedia Broadcast Supplement for Public Warning Systems, mission-critical video and data services, LTE support for Vehicle-to-Anything (V2X), latency reduction, high power LTE for certain bands, channel model above 6 GHz and robust call set-up for Voice over LTE (VoLTE).

By the second half of 2017, the focus of 3GPP work will shift to Release 15 in order to deliver the first set of 5G standards. For instance, the importance of forward compatibility in both radio and protocol design was stressed. Its functional freeze date including stable protocols would be on September 2018, thus the timeframe for a commercial deployment will be at the end of the decade.

One of the most remarkable proposals for the definition of 5G is the utilization of Filter Bank Multicarrier (FBMC) modulations instead of the well-known Orthogonal Frequency-Division Multiplexing (OFDM). The next are the most important advantages offered by FBMC with respect to OFDM for the railway environment:

- FBMC offers higher bandwidth efficiency, which is very beneficial since the simultaneous communications between different trains can be more efficiently allocated into the scarce spectrum available in railway environments.
- Coexistence between the current GSM-R and the new broadband systems is a major concern in the railway industry. OFDM-based systems usually exhibit a high co-channel interference, leading to a potential performance impact on current GSM-R systems. FBMC-based systems are much more efficient, thus allowing for better coexistence with current systems.
- Improved multiple-access facilities in the UL: due to the use of close-to-perfect subcarrier filters that ensure frequency localized subcarriers, FBMC does not require sophisticated synchronization methods for avoiding multiple-access interference. Nevertheless, while OFDMA is suitable for allocating efficiently a subset of subcarriers per user in the DL, the situation is different in the UL, because user signals must arrive at the Evolved NodeB (eNodeB) synchronously, both in terms of symbol timing and carrier frequency. For a practical deployment, a close-to-perfect carrier synchronization is necessary, which is affordable in a stationary network, but becomes a very difficult task in a network that includes mobile nodes.
- Suitability for doubly dispersive channels: the waveforms used in FBMC can be optimized for doubly dispersive channels like the ones present in high-speed train communications, hence allowing for a compromise between time and frequency channel response.

However, there are some drawbacks. It must be noticed that channel estimation is more challenging in most FBMC schemes with respect to OFDM. Moreover, whereas OFDM offers full flexibility regarding MIMO structures, FBMC can only be used in certain MIMO schemes. Only schemes such as Filtered MultiTone (FMT) offer the same flexibility as OFDM, but FMT suffers from the same bandwidth loss as OFDM. Alternatives to FBMC such as Generalized FDM (GFDM) and Filtered OFDM (fOFDM) are also being considered as candidates for 5G systems.

For inter-car communications, IEEE 802.11p is planned to be deployed in smart cars in the near future. Therefore, IEEE 802.11p can be an option for inter-train communications if high data rates are not required. Other solutions based on UWB technology or mmWave solutions in the range of 60 GHz carrier frequencies are expected.

Furthermore, spectrum allocation is always a challenge. Industrial Scientific and Medical (ISM) bands at 2.4 and 5 GHz are always available but they imply potential problems, in terms of security. Additionally, there is some discussion on the possibility of using the Intelligent Transportation System (ITS) band at 5.9 GHz for urban rail systems [58]. Facing the problem from the business perspective, partnerships with mobile operators to deploy mobile networks and also to provide some non-safety services to operators and stakeholders is possible but implies some regulatory challenges that should be addressed.

### 4.2. Migration Roadmap

Recently, UIC has started the migration from GSM Phase 2+ to LTE ensuring that the life cycle of GSM-R will be extended. Some researchers point out that the coexistence between GSM-R and LTE-Railways (LTE-R) is expected to last years [42]. Furthermore, LTE migration is envisaged to move at different paces. Considering the lack of a global standard for Communications Based Train Control (CBTC), metro railway operations are likely to adopt LTE quickly, particularly in new lines. In main lines under international standards, the transition will occur probably in two phases. Initially, the non mission-critical services will be carried out by the LTE networks, while safety and mission-critical services and features will keep using legacy networks. Following the maturity of LTE, all services will be then gradually transferred [59]. At the moment that suppliers standardize ETCS on IP networks, LTE-R will replace GSM-R. A schedule of LTE-R deployment is shown in Table 9.

Before considering different scenarios for future communications in railways, a number of hypotheses have been proposed to determine where the changes to the current environment are likely, and may influence options for the future. These hypotheses consider the period relevant to the study (i.e., the next 15+ years) and are listed in Table 10. As it can be observed, communications and applications are the ones that are expected to evolve at a higher pace.

## 5. The Rise of the Internet of Trains

Long before IoT was coined, railway operators and infrastructure managers were actively using M2M technology and data analysis to improve the maintenance and performance of their assets. The IIoT has had a major impact on the transportation industry, with the advent of autonomous vehicles and improved cargo management. Nevertheless, although they may have been pioneers, the reality is that the rail industry has barely scratched the surface of what is possible. As IIoT continues to evolve, it is bringing greater standardization, openness, and scalability to the information provided to operators: they gain insight into how their assets are performing, which opens up many new possibilities to use big data in more creative and effective ways. Nonetheless, the fact that trains operate at such high speeds through tunnels and extreme weather conditions, presents real challenges when it comes to deploying IIoT systems.

Regardless of the challenges, IIoT has the potential to revolutionize the railway industry. A rail network comprises thousands, if not millions of components, from rolling stocks to signals, rails and stations. All elements need to work cooperatively. The Internet of Trains holds the promise that rail systems can leapfrog interoperability, safety, and security issues, while modernizing rapidly. It refers to the use of networks of intelligent on-board devices connected to cloud-based applications to improve communications and control systems. The same network that strengthens safety has enough capacity to deliver data that serves a variety of applications across the rail system to reduce costs and improve operations. The usage of IIoT is possible thanks to advances in the following underlying technologies:

- Telecommunications networks are becoming dedicated to IIoT applications and, as it was described in Section 2, broadband communications are getting inexpensive, faster, and ubiquitous. Train companies run fiber along their tracks and have relationships with mobile operators to use their networks to maintain continuous mobile connectivity. M2M technology can boost efficiency by using sensors embedded into different objects and systems to automate tasks and deliver real-time monitoring and analysis.
- Sensors for data acquisition are getting smaller, more affordable, and now consume less energy. In some cases, battery life can be extended to up to five years, which is important, because it is not always possible to be close to an electrical supply.
- Cloud-based services have become more pervasive, fueled both by fast connectivity and ever-smarter devices. They can be used to store sensor data and to provide the computation required for big data analytics.
- Big data and the Cyber-Physical System (CPS) enabled by Industrial IoT (IIoT) allow the different transportation modes to communicate with each other and with the surrounding environment, paving the way for truly integrated and intermodal solutions.

### Industrial IoT Developments in the Rail Industry

Renowned commercial companies have been investing recently in IIoT. Next paragraphs outline opportunities for the railway ecosystem (i.e., technology vendors and operators), including some maintenance and monitoring initiatives that today are making the smart railway ecosystem vision a reality.

Trenitalia’s Frecciarossa is working with SAP to develop a Dynamic Maintenance Management System (DMMS) [60]. The system presents cost savings between 8% to 10% of its maintenance bill. In this case, hundreds of sensors collect data in real time (from braking systems to the sliding doors), uploading them into SAP’s cloud every ten minutes. Trenitalia runs 8000 trains per day using a fleet of 30,000 locomotives, coaches, and freight cars. Once the data are in the cloud, they are analyzed using SAP Predictive Maintenance and Service software and they are processed by the predictive analytics tool SAP HANA. Thus, Trenitalia can build predictive models using machine learning and also trigger actions (for example, when engine temperature hits a particular threshold, to help keeping trains running without delays). Key metrics, and diagnostic and management data are accessible by engineers and are visualized in real-time: the number of trains that are out of service, alerts that imply a maintenance action, the status of trains on the track, or the number of passengers. DMMS will be fully up and running across all Trenitalia’s rolling stock in 2018 and it will generate a full petabyte of data annually. Next, Trenitalia is hoping to automate the few remaining parts of diagnostics and maintenance that cannot be spotted by sensors, such as the roof and undercarriage of the trains, which still need a visual inspection. In the future, these tasks will be automated using cameras, instead of the visual inspection required today.

In 2013, the Finnish state-owned railway company [61], in order to improve its competitiveness, started to embed sensors into its systems to monitor possible failures related to the weather conditions. Previously, such a company performed maintenance in two ways. First, there was a scheduled maintenance that affected the most critical systems (e.g., bogies and wheels). Due to this type of maintenance, parts were replaced even when they still could be used more time. The second maintenance procedure consisted of fixing parts after they broke down. This kind of maintenance could not be predicted easily and derived in missed routes and unsatisfied customers. In order to prevent the problems that arose from these two maintenances procedures, the company developed a predictive maintenance program that monitors the state of the most relevant parts constantly. This system estimates through mathematical models when a part is likely to fail, so that it can be replaced before to avoid unplanned downtimes. To optimize the time between maintenance events, the company analyzes the data collected through a Statistical Analysis System (SAS) and determines if critical elements like the turning wheels or the wheel-and-axle sets need to be replaced. With all the improvements carried out, the company estimates that maintenance work will be reduced by 35 %. Moreover, since the cause of a failure can be identified more easily, the reliability of the trains is enhanced and savings are obtained in terms of time and money. Furthermore, the knowledge obtained through the system allows the company to minimize their spare part stock, buying and keeping only what is predicted that will be needed in the near-future.

Predictive maintenance is also encouraged by Siemens together with Teradata [62]. They expect predictive maintenance will evolve towards next-generation maintenance, creating a whole new business model to provide completely new services with up-time guarantees, risk-sharing models, and performance-based contracts for mobility systems.

Another example is represented by the French SNCF [63], which is also using IIoT powered by IBM Watson’s deep learning analytics platform and SigFox’s IIoT network. These alliances are part of the company’s 2020 strategy to become an industrial leader striving for operational excellence and optimum efficiency.

SNCF has developed a prototype where data acquisition devices are fitted into the transmission system on a *Train à Grande Vitesse* (TGV). Data are transmitted over GSM-R and can be accessed remotely at the train depot, enabling technicians to see how well the gearbox is performing. SNCF also uses Sigfox communications devices to measure the water level tank in TGV toilets, what speeds up the turnaround time when the train arrives at a depot. Besides, engineers can connect to running trains in real-time, enabling SNCF to figure out whether a component is likely to fail, which could lead to the train being taken out of service. The cloud enables SNCF to run distributed calculations, whose results can be reinjected into its train and rail maintenance processes.

## 6. IoT-Enabled Services: From More Efficient Operations to New Business Models

**Figure 3:**
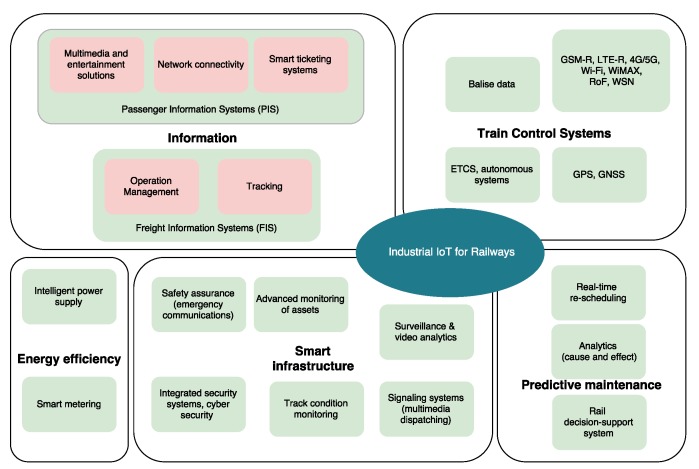
Industrial IoT-enabled services relevant to the rail industry.

Legacy infrastructure is gradually being replaced by Train Management Systems (TMS), which transform trains in communications hubs that exchange data among them and with network control centers. Moreover, M2M communications allow operators to optimize and make safer use of equipment and infrastructure. The following subsections describe examples of IIoT-enabled services (a general vision is shown in Figure 3).

### 6.1. From Reactive to Predictive Maintenance

It is expected that maintenance costs will rise in the next years due to the aging of the infrastructure and the increasing number of passengers and freight transported. Due to this trend, there is a demand for monitoring complex maintenance operations related to the different elements that conform the railway system [64].

Maintenance decisions about critical items of infrastructures can be improved by using the precise location of the train, its speed, weight, data from vibration sensors located alongside the track, weather reports, and details on how long the power connector is disconnected from the catenary during operations [65]. The fusion of this information with other meta-data, such as catenary dilation factors and track temperature, can further enhance the decision-making process and help to create more sophisticated rail scheduling software. For instance, Firlik et al. [66] studied how to monitor the state of light rail vehicles and their tracks. The researchers made use of sensors embedded into the axle boxes to adapt dynamically maintenance requirements and speed limits.

For instance, in [67] it is analyzed how to schedule preventive maintenance depending on different strategies. Furthermore, in [68] the authors studied the application of big data techniques to make decisions on the maintenance of railway tracks. A similar approach is described in [69,70], where the authors propose the use of an expert and a Decision Support System (DSS) to plan and schedule different common rail activities.

In recent years, ballasted tracks have been replaced by systems based on slabs, which are more secure and sustainable in high-speed railways. Preventive maintenance can be carried out in such infrastructures by embedding sensors that track movements and vibrations [71,72]. The design of such systems has to consider large areas in remote places that have no Internet access or electricity. In such scenarios the information collected is sent to passing trains that transmit the data later to a Remote Control Center (RCC). Another research article focused on the evaluation of the influence of train vibrations is presented in [73]. In the case of [74], the system proposed includes threat detection. For instance, Sa et al. [75] present a shape-based method for analyzing the normalized electric current patterns in Railway Point Machines (consisting in a motor, reduction gear, several bearings, derive-detection rods, and switches) in order to detect the replacement conditions with acceptable accuracy.

Ngigi et al. [76] describe the benefits of using predictive control systems to monitor different activities. Such a kind of systems is used for making better decisions when determining maintenance procedures. Specifically, there is a type of algorithms called Model Predictive Control (MPC) that has been devised explicitly for monitoring actions related to certain assets in order to anticipate events. The complexity of the vehicle dynamics usually involves using approaches like extended Kalman filters, which are able to estimate the dynamic performance of certain elements as the train moves, or particle filtering, which can assess the state in non-linear non-Gaussian scenarios in order to detect imminent faults. Furthermore, Ngigi et al. point out that Wheelset Condition Monitoring (WCM) systems can be applied to estimate the deterioration of the wheels through different sensors and automatic identification systems. Finally, the authors conclude that, in order to monitor the conditions in high-performance real-time scenarios, it is required to make use of WCMs and simulation techniques.

Another example of DSS is presented in [77]. Saa et al. propose a smart tool that helps railway infrastructure designers to create more secure and more efficient electrification systems. The tool prevents dangerous situations associated with errors during the design stage. The main novelty of the article is its holistic approach, which includes all railway systems in order to optimize infrastructure design through a knowledge system based on rules that incorporate expert knowledge. However, the researchers indicate an obvious disadvantage: the tool depends on the information received from the railway companies, which are usually not very collaborative among them. It is expected that this problem will be tackled in part by the European initiative for railway interoperability.

#### Key Findings

IIoT can increase the safety and efficiency of the rail traffic by providing preventive maintenance. Economic savings can also be obtained through the simplification of processes and making better decisions by using analytics based on sensor fusion of the data collected from trains and other infrastructures. Information concerning the categorization of faults can be analyzed across multiple assets, even multiple operators, to spot trends and identify areas for preventive maintenance. Additionally, data analytics can speed up root cause analyses, reducing labor time.

It is also required to increase the level of automation on maintenance procedures and other routine tasks, like ballast replacement, tamping, and track relaying. For example, remote monitoring helps to reduce maintenance time in the train depot. Another example can be train windshield water tanks equipped with a level sensor. Thus, a technician is able to access this information via a web application on a tablet to see whether the water needs topping up. The amount of data that has to be collected requires high-capacity wireless train-to-ground communications.

The following are a list of improvements that can be achieved:

- Increased up-time through a significant reduction of unplanned downtime.
- Extension and flexibility of maintenance intervals because the risk is understood.
- Improved utilization of assets (e.g., more mileage with fewer cars).
- Enhanced planning, with streamlined Supply Chain Management (SCM).
- Maintenance can be performed at the least costly location. IIoT will have an important role in applications for dynamic maintenance as a provider of additional sources of data collected by sensors. In this way, in a Computer Integrated Manufacturing (CIM) context, an Enterprise Resource Planning (ERP) will act as an ad-hoc software extension that will manage the collected data.
- Uptime guarantees can be provided.
- Increased service contract capture rate, recurring revenues, and higher percentage of the total service revenue.

### 6.2. Smart Infrastructure

Infrastructure monitoring (bridges, viaducts, tunnels, rail gaps, frozen soil, leaky feeders) can provide significant benefits in different aspects like efficiency or safety.

Furthermore, the lack of safety and security monitoring of railway infrastructure increase the risk of train collision, derailment, terrorism and failures in the wagons. For instance, with 35% of train delays still caused by infrastructure or rolling stock failures, this is one obvious area where IIoT could offer vast improvements in performance.

#### 6.2.1. Advanced Monitoring of Assets

**Figure 4:**
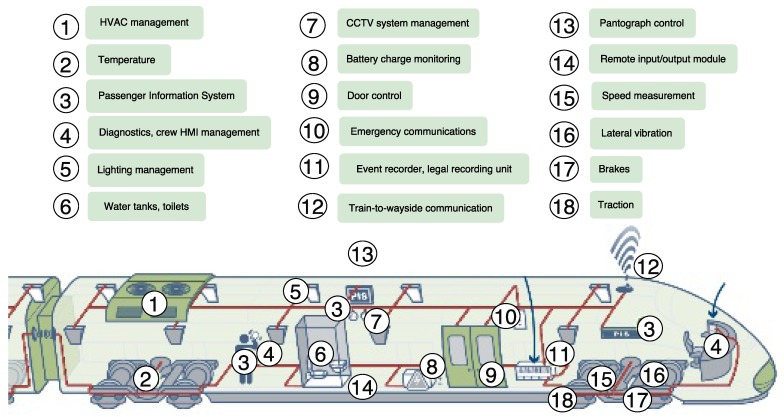
Systems usually monitored in a train.

Sensors can be used to decrease the failure rate and enhance the reliability of trains, signals, and tracks. Such sensors are able to monitor equipment with the objective of generating alerts about the need for attention on critical elements of the train. In this way, costs are decreased and asset usage is optimized by lowering the number of trains taken out of service for inspection, preventive maintenance, or for replacing certain parts after a deficiency is detected. The most common systems to be monitored are represented in Figure 4.

As it was previously explained in Section 2.6, infrastructure is usually monitored by using WSNs, which are able to assess the condition of tracks, track beds, bridges and the equipment placed on the tracks. Moreover, WSNs can be used to monitor tunnels or to detect intrusions and abandoned items in stations. It is worth mentioning Structural Health Monitoring (SHM), which is currently an essential field for the railway industry [78] and which has been reviewed recently in the literature [79]. Traditionally, SHM systems made use of sensors wired to data acquisition systems, but, thanks to the evolution and the lower cost of wireless devices, in recent years researchers have proposed solutions based on WSNs [80]. A relevant requirement is the need for a precise time synchronization with a resolution of microseconds [81]. This requirement is due to the fact that certain measurements, like vibration monitoring, demand accurate timing and synchronized sensing at high sampling rates [82].

The reflections derived from the metallic structures of the train also pose a problem for WSN communications due to the multipath effect. This issue can be addressed by modeling the communications channel and then selecting a proper physical layer. Different researches have studied this problem in different SHM scenarios, including bridges [83] and railway tracks [84]. Another example of a WSN deployment at railway tracks to analyze the vibration patterns caused by trains is described in [85]. Moreover, existing monitoring methods are studied in [86], where the authors integrate three methods to monitor rail damage in the turnout (railroad switch) zone (i.e., fiber optic detection systems, optical imaging and Lamb guided wave detection systems).

WSN-based condition monitoring for the rail industry is the object of the survey presented in [87]. Such a review analyzes the most commonly used sensors for condition monitoring, their configuration and the main network topologies. Another interesting WSN-based system for condition monitoring is proposed in [88], where it is collected data on the infrastructure structural health during the trip to later send the information to a BS. Then, such a BS makes use of the next trains as data mules to upload the information.

Another real-time WSN-based railway SHM system is presented in [10]. In such an article the researchers focused on designing a customized MAC layer and a synchronization algorithm. According to the results, the sensor nodes that belonged to the same BS presented jitter values within 1 μs, while nodes from different BSs had a maximum jitter of 2 μs. It is also interesting the work of Li et al. [89], who describe models and algorithms for optimizing the physical topology of a sensor network aimed at monitoring the condition of the infrastructure.

A railway bridge is monitored by a WSN-based solution in [90]. In such a system eight nodes collect data that are transmitted to a TmoteSky that acts as BS. The system uses accelerometers to detect when a train approaches and crosses the bridge monitored. The system uses a self-organizing routing protocol whose objective is to make the data reach the BS, which sends them to the RCC through a UMTS transceiver. Other authors proposed similar WSN-based systems for monitoring railway infrastructure either by using Wi-Fi [91] or Zigbee [92].

#### 6.2.2. Video Surveillance Systems

These systems are able to show high-resolution images or videos within the train (onboard, during the operation of the train), along the tracks (for the advanced monitoring of assets) or in the station [93].

Intelligent CCTV cameras not only provide a record of events in case of an incident, but actively provide real-time alarms on the occurrence of potential problems, allowing for obtaining timely intervention responses and potentially reducing service outages. Moreover, if video recording are required by a law enforcement agency, there is no need to send personnel on-board to obtain the hard drive manually. The images collected can be either stored in a local server or transmitted in real-time to TCCs.

In the past, operators detected the lack of a surveillance on-board solution, particularly because of the absence of broadband wireless communication systems between trains and the control center. Moreover, there are just a few examples in the literature that study video surveillance in railways. An example is the Security Management System proposed by Bochetti et al. [94], who integrated devices for access control and surveillance. Their work also includes the development of a security platform that uses a middleware to embed heterogeneous sensors and that is able to adapt QoS requirements to the priority of the data transmitted.

A video surveillance platform deployed in subway of Beijing (China) is presented in [95]. The development includes different modules that allow the platform to manage different elements of the system, setup alarms, detect failures automatically or visualize all the data on a GIS map.

Apart from the subway lines, there are few CCTV systems for conventional trains and high-speed trains due to the lack of effective wireless train-to-ground communications. An exception of such systems is the one proposed by Flammini et al. [96], which is aimed at correlating data from different sources like environmental sensors, intrusion detection sensors, positioning systems, identification systems, video-surveillance devices and even from CBRNE (Chemical Bacteriological Radiological and Nuclear) sensors. Regarding this last type of radioactivity sensors, they are deployed for preventing attacks based on the so-called dirty bombs. To detect explosives, there exist specific devices usually installed near the turnstiles that can detect evidence of gun powder on the hands and clothes. Furthermore, to detect weapons and explosives on passengers, it is currently under research the use of terahertz cameras.

#### 6.2.3. Operations

The same infrastructure put in place to provide safety applications can also be used for non-safety applications in order to leverage the investment made by the operators.

Subways and suburban rails can make use of the data sent by trains to indicate the customers through smartphone applications when such trains are scheduled to depart, arrive, or if there are delays. Additionally, IIoT can modify current railway business models: the use of analytics based on the usage determined by sensors may transform providers from sellers to leasers of equipment. This change yields constant sources of revenue for the providers, while altering the expenses of the operators from CAPital EXpenditure (CAPEX) to Operating Expenditure (OPEX).

A remarkable example of operations enhancement is the design of an Electric Multiple Unit (EMU) IIoT-system presented in [97]. It is oriented to the Maintenance, Repair and Operation (MRO) of high-speed trains in China. Massive seamless embedded RFID tags and sensors in train-ground transmission networks are able to perceive the status of high-speed trains in real-time, using holographic train visualization and delivering transit alerts. The use of multi-source and multi-level raw data in maintenance and repair processes, collecting various production aspects, such as train flow, part flow, labor flow, and equipment, helps to monitor productive processes and logistics during the whole life-cycle. This study is expected to increase 25% the productive output of EMU operation-level maintenance and 20% the overhaul-level maintenance which includes dismantle, repair and assembly.

Nowadays, railway Information and Communications Technology (ICT) system implantation is growing, while the deployment of DSS based on the data collected is still emerging. There are currently interoperability issues among the different systems, what derives into missed opportunities. The use of models based on semantic data is one of the ways to improve interoperability, since they allow for an easy integration of data coming from diverse sources. For example, Briola et al. [98] use ontologies and natural language interfaces to handle the information collected from a traffic control center. Moreover, Tutcher et al. [99] study the most relevant design patterns to provide extensibility and interoperability, and introduce the concept of Asset Monitoring As A Service (AMaAS).

Operation scheduling is a complex problem that is influenced by multiple factors, like track capacity or travel distance. Some researchers have proposed its modeling through solutions able to create line plans by specifying different parameters, like the train capacity, passenger demand, the line frequency, the number of transfers or the stopping patterns [100]. The interested reader can find further information in [101,102,103,104,105], where the problem of real-time scheduling is considered under different approaches.

#### 6.2.4. Key Findings

The railway industry can benefit from the WSN ability for carrying out easy deployments that can reach places where a wired solution may not be installed. Additionally, such deployments can harness the flexibility, scalability, and self-organizing capabilities of WSNs. Nevertheless, WSNs have to deal with several issues specific for railway environments, like communications reliability, the necessity for higher sampling rates for measuring fast changing dynamic signals (e.g., vibrations), fast transmission rates, the capacity of managing high-volumes of data, energy efficiency, energy harvesting and the possibility of managing heterogeneous sensor signals (data fusion).

Regarding video surveillance systems, the existence of a real-time viewing mode, a record mode, and a search/playback mode allows security managers to avoid threats. These systems support video analytics, intelligent incident response, and emergency communications. Cameras can increase passenger safety and protect assets by integrating video surveillance systems across a network infrastructure. By integrating heterogeneous subsystems (environmental and intrusion detection sensors, positioning and identification systems) and potentially thousands of cameras, an overall view of the whole infrastructure (i.e., trains, tracks, depots, and stations) can be obtained by operators and management systems at the TCC or by the staff operating in the field, with video analytics and real-world maps identifying, locating, and recording threats.

Train delay is one of the most relevant factors that affect the perceived quality, since it affects the capacity of the system, its punctuality, its reliability and even its safety. Moreover, the availability of precise train positioning is essential for setting routes, controlling the traffic, rescheduling, and for offering accurate information to the passengers and the maintenance operators. Thanks to the transmission of real-time positioning data to control centers, the systems embedded into the train can help to reduce congestion by optimizing the deployment of the equipment and managing the track capacity.

### 6.3. Information

Railway industry is also currently challenged by the improvement of the experience of the passengers and the management of the freight. On the one hand, passengers usually demand better train punctuality, precise scheduling information and improved on-board entertainment. On the other hand, logistics often require cost-effective solutions that include the whole monitoring of the freight. Thus, taking the characteristics of the information into consideration, two types of targets have to be distinguished: passenger and freight.

#### 6.3.1. Passenger Information System (PIS)

PIS is a key communications link between operators and passengers. PIS represents an electronic operating tool that provides, at any given time, visual and acoustic information to passengers on a route, both automatically or programmed manually. PIS includes real-time train tracking, route information and scheduling, travel planning, passenger infotainment (real-time HD video for entertainment or business, video conference, live broadcast), and online connectivity solutions. Along with system safety and reliability, the ability of the operators to provide accurate and useful information (i.e., departure/arrival times), and more comprehensive services, as well as the feeling of being in control or participating, is a key component of passenger satisfaction.

PIS architecture spans across three different environments: rails, fixed installations such as stations and depots, and a centralized control center. This architecture is shared with the security, control and monitoring, and network functionalities. A wireless or wired connection is used for communication between the display device, the station computer, and the main server. The current position of trains is transferred to the relevant computer stations through the main server, where data are displayed, and new data for next stops can be calculated. The TCC is used for controlling and monitoring the trains.

A PIS example could be a trip planner application that could recommend the fastest or most comfortable trip, showing live train times, available car parking or passenger loading. Passengers will make informed choices about what option will provide them with the best experience according to their personal preferences (i.e., whether it is more important to have the shortest trip time, or to reserve a seat). The inclusion of historic data will enable the evaluation not only for a current trip, but also in a predictive way for a trip planned in the future.

The combination of passenger loading information from trains with social networking applications will help to spread demand peaks [106]. For example, this can be achieved by offering the most efficient passenger exit considering the loadings of other inbound trains. In the case of interoperable tickets (valid for metro, buses, and bikes), intermodal travel could be encouraged by providing seamless connections to other modes [107].

Moreover, fusing status information from diverse on-board public-facing assets such as toilets, chillers and ovens, and presenting it to service organizations with current positional information, can improve the customer experience and reduce the penalty costs associated with having these assets out of service. Toilets can be automated to reduce costs and provide better service to the passengers. Currently, most operators are no able to determine in real time the state of toilets without performing a manual checking. Regarding the food, it could be replenished at a station if information about the items sold are available in real time. Furthermore, to avoid problems with the refrigerators, which cannot be in service constantly, temperature might be monitored and controlled remotely. Traditional hand-held ticketing systems are being updated slowly to more sophisticated solutions that alleviate the crowdedness and ease the passenger trip. Furthermore, considering that several smart cards used in public transportation present faults [39,40], innovative solutions are emerging, like electronic ticketing systems that use QR codes, RFID and NFC [108], and even ticket-free solutions, like the pilot, due to begin in July 2017, between the UK-based rail operator Chiltern Railways and the travel technology company SilverRail Technologies [109]. Such a pilot will use Bluetooth sensors that activate an geolocation tracking app used to open ticket gates and determine the trips performed. In this case, the customer is billed at the end of the day with a best-value guarantee ensuring they are charged the appropriate fare for their trips. Note that in order to provide security to the ticketing system, dedicated communication lines should be deployed.

#### 6.3.2. Freight Information System (FIS)

Railways offer an alternative for freight transport that has low external costs and a reduced environmental impact. In fact, trains consume less energy and emit less CO${}_{2}$ than the other means of transport by road, air and water. However, currently there are legal, operational and technical constraints that reduce its capacity and performance. Moreover, the reliability of this specific services need to be improved. The modal share of rail transport is modest, with rail accounting for 11% transportation in Europe, and 6% of intra-European passenger transport according to reports of the EC in 2014. There are two major challenges that have to be faced. First, there has to be created a new specific profile aimed at on-time deliveries. Second, a growth of productive capacity and an increase of cost competitiveness by addressing current challenges, such as interoperability, the optimization of existing infrastructure, and the promotion of synergies from other sectors.

FIS delivers real-time information on freight traffic to provide a significant picture of freight transportation movements, effectiveness, and planning. FIS is subdivided into two solutions: operation management solutions for capacity and freight management, which ranges from booking to rolling stock planning; and tracking solutions for real-time location information of cargo containers. FIS helps freight operators to make infrastructure and planning decisions based on robust, reliable, and consistent data.

Several research articles have dealt in recent years with freight trains. For example, Scholten et al. [110] focused on monitoring their integrity. In [111] it is presented another system for the transport of dangerous materials by rail. In such an article it is evaluated the generation of business rules from a semantic knowledge system using the information collected about different elements and parameters of the rail system.

Among the numerous elements to be monitored, rolling bearing is specially interesting, since it is used in freight trains and it is considered an important part whose fault can affect train safety. Infrared and acoustic monitoring techniques have been tested for monitoring the rolling bearing, but they have disadvantages, like the detection of false positives. Additionally, on-board monitoring solutions are not useful in freight trains, because cars are not attended and there is not a constant power supply. Nan et al. [112] propose a WSN-based solution for freight trains that allows for monitoring rolling bearing through accelerometers, which are actually used to measure the vibrations. Another application for the monitoring of freight trains transporting hazardous materials is presented in [113,114]. The application uses a WSN to measure environmental parameters using heterogeneous sensor technologies.

Tunnels, bridges and highway crossings are examples of the elements that can be encountered by a train on its way. The most common problems that occur in these scenarios are due to differences in substructure and loading conditions. For instance, if the track is deformed substantially at these points, the dynamics of the train change, what eventually derives into a deterioration of the structural elements. The identification of the factors that contribute to this deterioration, as well as its mitigation through maintenance procedures, are essential for safety and economic reasons. For instance, Tutumluer et al. [115] evaluated track transition performance in different high-speed scenarios, analyzed the reasons behind the deteriorations and proposed diverse methods for improving track performance.

Rail freight operations planning together with revenue management has not been reported generally in the literature. Crevier et al. [116] present a new bi-level mathematical formulation that combines both pricing decisions and network planning policies (e.g., car blocking and routing as well as the assignment of blocks to trains and scheduling). Besides, Bilegan et al. [117] propose a strategy for increasing revenues by accepting or rejecting transportation requests in order to accommodate the future foreseen demands with higher potential profits. In [118] it is presented a model for evaluating the decisions taken in inter-modal transportation that includes the contributions related to operators, providers and users. The optimal policy is characterized by Luo et al. [119], showing how dynamic forecasting coordinates capacity leasing and demand acceptance in intermodal transportation. Furthermore, Wang et al. [120] studied how to optimize the benefits of container transportation operators by allocating resources when the capacity and the effects on the network are unknown. Finally, other authors focused on specific applications, like Masoud et al. [121], who analyzed the optimization of sugarcane rail transport systems.

#### 6.3.3. Key Findings

PIS tools improve passenger experience while allowing for offering informed choices, determining the status of main facilities or using innovative solutions of smart ticketing. A FIS also improves the labor utilization and productivity, and nowadays is widely adopted by logistics companies for better customer support and loyalty. The following are the main advantages of FIS for railways: improved dynamic train performances; real-time information provision, which is especially important in the case of hazardous goods and to plan revenue management; it enables the intercommunication and exchange of information from train-to-ground; and remote real-time diagnosis using sensors embedded into wagons.

### 6.4. Train Control Systems

#### 6.4.1. Autonomous Systems

There are two types of autonomous systems: semi-automatic and fully autonomous. The former are related to operations like signaling and train braking systems. The latter make use of artificial intelligence techniques like genetic algorithms and fuzzy logic. There are not many references in the literature about fully autonomous trains and most are focused on subways or light rail systems [122].

#### 6.4.2. Safety Assurance and Signaling Systems

The improvement of the safety is one of the major goals when applying IIoT in railway environments. For instance, an accurate on-board positioning system is essential in order to determine the position of other trains and then avoid collisions, perform safer operations in close proximity and optimize the use of the tracks. Another safety application is related to the measurement and control of the speed [123]. There are currently systems that show the train speed to the drivers and later report it to central control systems. Some of such system are able to interact with wayside signaling systems with the objective of regulating the train velocity and they are even able to stop the train completely if certain conditions are met.

There are four major systems where automation and IIoT can bring significant advantages: signaling, level crossing control, interlocking, and dispatching.

Signaling systems can adjust remotely the speed and braking of the train. Signaling systems are usually equipped with RFID devices that are embedded into the tracks, but wireless ground-to-train signaling is becoming habitual. Most of the new European lines are equipped with ETCS level 2, as it was explained previously in Section 3, which requires train-to-ground communications.

Level crossing control has also a huge impact on safety. According to ERA, in 2010 more than 300 people died in incidents that took place at level crossings, representing 30% of all the deaths related to European railways. IIoT can help to decrease those statistics by deploying cameras and sensors for increased safety. One example in the literature relying on video is presented in [124]. Other alternatives use Ultra-Wide Band (UWB) technologies [125].

Interlocking works together with the signaling system to avoid collisions at crossings and junctions. It essentially makes use of traffic lights and signals that prevent trains from moving forward if a scenario is not safe. IIoT enables the automation of the interlocking system and enhances it by integrating the data received from the signaling system.

Furthermore, comprehensive dispatching information including text, data, voice, image, and video, can be provided by drivers and yards to the dispatcher. Supporting functionalities such as voice trunking, dynamic grouping, temporary group call, short messaging, and multimedia messaging, is also needed. For instance, in case of automatic driving, dispatching video stream of doorways is required to ensure that doors are clear prior to the train departure.

The data collected from on-board and wayside embedded devices provide a large amount of information that can be exploited through data mining techniques, which allow for the identification of structural patterns that cannot be discovered easily. Several researchers studied the application of such techniques in railway scenarios. For example, Goverde et al. [126] exploited the information from the describer records of a train to evaluate potential conflicts associated with the scheduling or the capacity of the railway system. Furthermore, such information is also used in [127] to develop a model for predicting the timing of certain events. The same authors from the previous reference also assessed the viability of using data mining techniques to exploit rail data like business processes [128]. Similarly, in [129] different real-time techniques are studied, but for controlling train traffic.

Recently, the authors of [130] proposed the use of fast scheduling and routing metaheuristics for managing train traffic in busy situations, taking special care of the control efficiency in conflictive traffic situations (e.g., multiple train delays). Furthermore, there is a recognized need for providing train locations in real time, which should be compliant with the railway safety requirements [131] (e.g., with the EN 50126 standard). Thus, some researchers evaluated the use of GNSS receivers for train positioning [132]. The results presented by the researchers show that, in a forest scenario, the GNSS-based system does not fulfill the requirements, and that it is required a sensor fusion structure composed by on-board positioning sensors. Therefore, a positioning system composed by a Doppler radar sensor and a GNSS receiver will meet the requirements.

A WSN architecture focused on secure railways is described in [133]. Such a system measures acceleration and makes use of ultrasounds to identify spoilage on railroads. Another system for detecting objects on the tracks is presented in [134], where the researchers apply image processing and electromagnets for the detection. Additionally, Wang et al. [135] use a WSN for early earthquake detection in high-speed railways that is able to send fast warnings to the control center.

#### 6.4.3. Cyber Security for Railways

As it was shown in previous Sections, rail systems have evolved significantly towards new technologies and communication-based systems led primarily by the technological progress. Despite the fact that security in the railway industry has been always related with operational safety, due to the increasing integration of ICT into land transport, mobile units and infrastructure, the number of potential cyber risks has risen steadily during the last decade. In the same way, train control systems are relying more and more on ICT systems and radio communications, even for the ones automated.

Cyber security is about protecting information systems against theft or damage, thus defending them against external and internal attacks and risks, in particular as a result of criminality. For this reason, future research related to rail security should focus on the rising of new cyber threats. For instance, the generalization of automation and computerization in the rail vehicles and signaling systems, could also become a high potential risk.

Every railway operator faces the massive challenge of protecting its own infrastructure reducing its vulnerability to cyber-attacks. In most cases, heterogeneous ICT technologies and software solutions are used and result in wide-ranging and diverse data sets. The protection of such environments is complex and multi-dimensional. A proper design of the architecture of the infrastructure will help to improve resilience, but it is essential to integrate safety into every aspect of the solution throughout its life-cycle. Cyber systems used on rail networks may be subject to unauthorized access through various means: remotely, via the Internet, or unsecured communication networks; through direct contact with infrastructure (e.g., through a USB port); locally, through unauthorized access to physical infrastructure, or an insider threat (e.g., infiltration).

Rail operators have to comply with a set of international industry and government standards on the topic of security (e.g., ISO 27001, NIST SP800-53, ISA/IEC 62443 or APTA). Nonetheless, each infrastructure and each security solution is unique. While meeting national and international security regulations, it is necessary a comprehensive analysis on how to design and protect information systems. It is also essential to develop, implement and maintain integrated solutions, and added-value services to protect sensitive information at any given time.

The most relevant vulnerabilities are related to weaknesses in control systems, information systems, system procedures, configuration and maintenance, software development, the communications network, or in the lack of training and awareness. All of them can be exploited by threats that can be originated by many sources, including:

- Connecting physical infrastructure (e.g., tracks, tunnels, bridges/viaducts, switches/rail junctions).
- Mobile units (e.g., locomotives, rolling-stock system).
- Train stations (e.g., exterior, interior or restricted areas) and areas outside the train station.
- Control systems (e.g., signaling, central and local rail traffic management).
- Communication systems and communication network.
- Power supply (e.g., catenaries, power supply, national grid, diesel stations).
- Staff (e.g., driving personnel, handling personnel, maintenance personnel, information processing personnel).
- Freight (e.g., non-dangerous, explosive, toxic, flammable).
- Passengers.

Regarding the classification of assets presented, access, construction techniques, control command and communication systems are considered to be the most vulnerable elements of the railway transportation systems. Indeed, these central elements are easily exposed to malicious uses leading to serious threats.

#### 6.4.4. Key Findings

Train control systems include signaling and safety assurance processes. Level crossing control, dispatching video stream or on-board positioning systems are examples of improvements. Furthermore, opportunities for research exist on scheduling and maintenance planning and on event prediction, among other new activities, considering autonomous and semi-autonomous operation. Cyber security is another field to be studied with more depth, since cyber risks are exacerbated by the enormous quantity of data resulting from the increasing number of devices, processes and services integrated. Even more, there are several sources of vulnerabilities, whose countermeasures have to be designed.

### 6.5. Energy Efficiency

IIoT can play an important role to promote energy efficiency taking the EU environmental, financial and regulation concerns into account. Moreover, the techniques required for optimizing energy efficiency are strictly related to other solutions previously described for tackling issues like advanced asset monitoring. However, note that up to four energy-efficiency levels can be distinguished in railway scenarios [136]: energy-efficient driving, the coordination and re-scheduling of multiple trains in real time, the creation of energy-efficient timetables, and energy-saving planning.

Regarding the coordination, Xun et al. [136] propose an autonomous system to coordinate trains by optimizing the time spent between contiguous stations. Before departure, every train is able to determine the optimum running time by estimating when the preceding train will depart from the next station. Thus, the system has to achieve a good balance between energy efficiency and the time waited by the passengers.

Energy efficiency can also be determined through smart metering methods. With a knowledge of the different consumers it is possible to perform an efficient energy management. Smart metering also allows for improving the management of assets and increases capacity. Three elements can be distinguished in smart metering systems: sensors, the communications between the different sensors, and the train-to-ground communications that require broadband links.

For instance, wheel bearings can be monitored through WSNs [137]. In such a paper two issues are investigated experimentally: propagation and energy efficiency. First, the electromagnetic wave propagation characteristics around a train for high signal reliability. Measurements show that the path-loss exponent is different depending on the scenario. In general, the path-loss exponent is lower on top of the train than beside the train. The value of the path loss exponent with narrowband at 434 MHz is on average 3.67 with antennas are located under the train, compared to 2.27 on top. The communications were evaluated during a five-week field trial onboard a train in bad weather conditions. The number of messages transmitted successfully per day was in average about 92%. The lost messages were due to fading or mechanical damages of the sensors. Second, energy scavenging for minimum maintenance of the sensor network was investigated. The researchers verified that the sensors could be powered by solar power. However, a theoretical study indicates that the most suitable method to power the sensors is energy scavenging by vibration.

The implementation of optimized train trajectories is also a topic under research. Speed profiles reduce energy consumption by avoiding running at reduced speed or unessential braking while arriving at planned times. An optimized train trajectory can be realized using a driver advisory system or Automatic Train Operation (ATO). Furthermore, the optimized trajectory needs input data, such as the train’s position, gradient, direction, speed and maximum speed, dwell time, and station locations. In [138] it is proposed a genetic algorithm for optimizing the train speed profile. The results obtained following the advice generated by the DAS when updating the system every meter, showed that the optimized trajectory could save energy up to around 25%. However, a train positioning system error under 100 m increases the energy consumption by less than 0.3%, while an error under 500 m increases it by less than 1.5% for uphill lines; and 1.3% and 5.2%, respectively, for a downhill line. These results imply that it is sufficient to locate a train through a GPS to save energy and that, for such a purpose, it is not necessary to make use of high-precision positioning data. Further research includes dynamic DAS to recalculate an optimized trajectory when a correction of the train location occurs in real-time. Additionally, a DAS connected to the TCC can be evaluated regarding the effect of train positioning errors when following an updated timetable, as well as the impact of such errors on the traffic management system. Bocharnikov et al. [139] examined the effects of varying the acceleration and braking performance in electrically powered suburban railways. Their solution makes use of a genetic algorithm that it is able to determine the optimal trajectory of the train from a set of simulations.

#### Key Findings

Energy-efficiency is cross-related to the other explained advanced services. For instance, WSNs can include energy scavenging capabilities for monitoring assets. Furthermore, additional energy savings and emission reductions can be achieved by considering the implementation of timetable optimizations (coordination and re-scheduling of trains in real time), the use of wayside devices for the storage of energy, smart metering methods, or energy-efficient driving (optimized train trajectories, enhanced vehicle comfort control and speed profiles).

### 6.6. Summary

**Figure 5:**
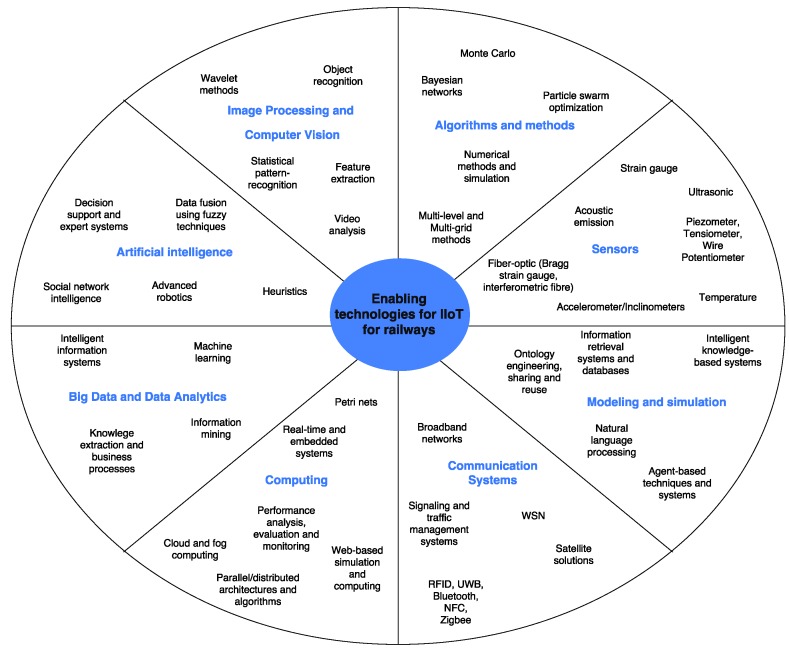
Enabling technologies for the IIoT of railways.

The main benefits explained in Section 6, summarized for the interested readers in Table 11, are just the tip of the iceberg and many other areas that could offer potential benefits have probably not even been identified yet. This table serve as reference to compare the different scenarios, objectives, technologies and architecture of the most relevant systems. However, please note that, due to the diversity of the systems analyzed, a straightforward comparison of experimental results would not be fair. Indeed, as it can be seen in Figure 5, enabling technologies, massive data aggregation, correlation, and analysis using highly-sophisticated algorithms have the potential to change operations, maintenance, yield management, and even passenger services in the future. As it was shown in this comprehensive survey, the IIoT is set to revolutionize train operations, enabling to improve customer service and the competitiveness of trains.

## 7. Conclusions

This survey examined the role of enabling technologies to revolutionize the railway industry. Broadband technologies, like LTE, provide the capacity needed to create novel services. A formal analysis regarding GSM-R requirements and services was presented to provide an understanding of future customer needs. LTE Release 11 includes the first feature for public safety (i.e., high-power UE). Starting from LTE Release 12, the standard adds characteristics such as IMS emergency calls, ProSe, PoC, GCSE, and eMBMS that will evolve LTE/LTE-A to be used as part of a broadband public safety network. LTE Release 13 includes the first set of specifications for mission-critical scenarios including MCPTT, enhancements of ProSe and GCSE, and the isolated E-UTRAN operation. Although the feasibility of LTE in the railway environment is evaluated, the deployment of a brand-new ecosystem will also require the design of a thorough migration strategy. In addition, WSNs constitute an essential part of the protection of the infrastructure, and M2M technology can boost efficiency by using sensors embedded into objects and systems to deliver real-time analysis and monitoring while enabling automation.

The fast pace of ICT technologies (e.g., cloud computing and big data) and communication networks enable the adoption of Industrial IoT to integrate the thousands, if not millions of components, from rolling stocks to the station. The Internet of Trains paradigm holds the promise that rail systems can leapfrog interoperability, safety, and cyber security issues, while modernizing rapidly. It refers to the use of networks of intelligent on-board devices connected to cloud-based applications to improve communications and control systems. The same network that strengthens safety has enough capacity to deliver enhanced data that serves a variety of applications across the rail system to reduce costs and improve operations.

Furthermore, the adoption of the paradigm opens a wide area of short- and medium-term potential applications. Examples like predictive maintenance, smart infrastructure, advanced monitoring of assets, video surveillance systems, railway operations, Passenger and Freight Information Systems (PIS/FIS), train control systems, safety assurance, signaling system, were detailed in order to expose the IoT capabilities to reinforce competitive advantages, to create new business models, and to change railways. For each of the services, the latest technologies and the main academic and commercial developments were thoroughly examined.

After all the analyses performed, it can be stated that the Internet of Trains and IIoT still face many challenges, such as standardization, interoperability, scalability, energy efficiency and cyber security, which would have to be addressed by researchers that will have to cope with the additional issues posed by railway environments and the specific nature of the operations and the networks.

## Figures and Tables

**Table 1:** Main characteristics of the different line types.

Characteristics	Urban	Urban/Inter-City	Inter-City	High-Speed
Maximum speed (kph)	s ≤ 70	70 < s ≤ 160	160 < s < 250	≥250
Line length (km)	l ≤ 20	20 < l < 100	100 ≤ l < 250	l ≥ 250
Parallel tracks (units)	1	2	3	4
Rolling stock	Single	Similar	Mixed	Very Mixed
Stock types	1	2–4	5–8	9+
Train stations	1–5	6–20	21–50	51+
Operators	1	2	3–5	6+
Passengers (per km of line)	*n* < 100,000	100,000 ≤ *n* < 200,000	200,000 ≤ *n* < 500,000	*n* ≥ 500,000
Range of services	Single	Small diversity	Multiple variances	Extremely varied

**Table 2:** Main characteristics of the most popular communication technologies for railways.

Parameter	GSM-R	P25	TETRA	802.11	WiMAX	UMTS	LTE-R	RoF	LCX	Satellite	FLASH-OFDM
Frequency	DL: 921–925 MHz, UL: 876–880 MHz	700 MHz	400 MHz	2.4/5.8 GHz	2.4/2.5/3.5 GHz	800/910 MHz, 2.1 GHz	450 MHz, 800 MHz, 1.4 GHz and 1.8 GHz	Variable	Variable	Limited	450 MHz
Channel bandwidth	200 kHz	12.5 kHz	25 kHz	20–40 MHz	1.3–20 MHz	5 MHz	1.4–100 MHz	10–100 MHz	30–1000 MHz	>20 MHz	1.5–5 MHz
Peak data rate	172 Kbps	40–100 Kbps	5–10 Kbps	>10 Mbps	>30 Mbps	>2 Mbps (stationary) >384 kbps (mobile)	DL: 50 Mbps, UL: 10 Mbps	1–10 Gbps	1–10 Mbps	>2 Mbps	DL: 5.3 Mbps, UL: 1.8 Mbps
All-IP in native mode	Not standalone	No	No	Yes	Yes	Yes	Yes	Yes	Yes	Yes	Yes
Handover mechanism	Standard	Standard	Standard	Proprietary	Standard	Standard	Standard, soft (no data loss)	Standard	Standard	Variable	Proprietary
Modulation multiplexing	GMSK TDMA	4FSK	DPSK TDMA	QPSK, QAM	BPSK, QPSK, 16-QAM	PSK	QPSK, 16-QAM and 64-QAM (OFDM, SCFDMA)	QPSK, 16-QAM (OFDM)	Std. and OFDM	FSK-PSK	OFDM
Maturity	Mature	Mature in US	Mature	Widely adopted	Mature, lead to WiMAX 2	Mature	Emerging	Concepts like ’moving cell’	Mature (N700)	Mature but costly	Mature
Market support	Until 2025–2030	US	Almost obsolete	Yes	Decreasing support	Moving to LTE	Building standards	Mature	Japan, Europe	Europe (Thalys, SNCF)	Flarion

**Table 3:** Comparison of the different WSN technologies. Color meaning: green (fully compliant with railway requirements), yellow (partial fulfillment) and red (non compliant).

Wireless Technology	Robustness	Real-Time Performance	Range	Link Throughput	Network Scalability	Power Awareness
IEEE 802.11						
IEEE 802.15.4						
Zigbee						
Zigbee Pro						
IEEE 802.15.1						
Bluetooth						
WirelessHART						
ISA 100.11a						
WISA						

**Table 4:** Services to be supported according to the radio type. Note that Mandatory for Interoperability (MI), Mandatory for the System (M), Optional (O) or Not Applicable (NA) [43,48].

Service Group	Type of Service	Cab	ETCS Data Only	General Purpose	Operational	Shunting
Voice-Call	Point-to-point	MI	NA	M	M	M
	Public emergency	M	NA	M	M	M
	Broadcast	M	NA	M	M	M
	Group	MI	NA	M	M	M
	Multi-party	MI	NA	O	O	M
Data	Text message	MI	NA	M	M	M
	General data applications	M	O	O	O	O
	Automatic fax	O	NA	O	O	O
	ETCS train control	NA	MI	NA	NA	NA
Specific features	Functional addressing (FA)	MI	NA	M	M	M
	Location dependent addressing (LDA)	MI	M	O	O	O
	Direct mode	NA	NA	NA	NA	NA
	Shunting mode	MI	NA	NA	NA	M
	Multiple driver communications within the same train	MI	NA	NA	NA	NA
	Railway emergency calls	MI	NA	O	M	M

**Table 5:** GSM-R call set-up time requirements [43,48].

Call Type	Call Set-Up Time
Railway emergency call	<4 s (M)
High priority group calls	<5 s (M)
Group calls between drivers in the same area	<5 s (M)
All operational and high priority mobile-to-fixed calls not covered by the above	<5 s (O)
All operational and high priority fixed-to-mobile calls not covered by the above	<7 s (O)
All operational mobile-to-mobile calls not covered by the above	<10 s (O)
All other calls	<10 s (O)

**Table 6:** Main GSM-R QoS requirements.

Requirements	Value
Connection establishment delay of mobile originated calls	<8.5 s (95%), ≤ 10 s (100%)
Connection establishment error ratio	$<10^{-2}$ (100%)
Connection loss rate	$<10^{-2}$/h (100%)
Maximum end-to-end transfer delay (of 30 byte data block)	≤ 0.5 s (99%)
Transmission interference period	<0.8 s (95%), <1 s (99%)
Error-free period	>20 s (95%), >7 s (99%)
Network registration delay	≤ 30 s (95%), ≤ 35 s (99%), ≤ 40 s (100%)
Call-setup time	≤ 10 s (100%)
Emergency call-setup time	≤ 2 s (100%)
Duration of transmission failures	< 1 s (99%)

**Table 7:** System characteristics of GSM-R and LTE-R.

Parameter	GSM-R	LTE-R
All-IP in native mode	No	Yes
Frequency	DL: 921–925 MHz, UL: 876–880 MHz	450 MHz, 800 MHz, 1.4 GHz and 1.8 GHz
Bandwidth	0.2 MHz	1.4–20 MHz
Modulation	GMSK	QPSK and 16-QAM
Peak data rate	DL/UL: 172 Kbps	DL: 50 Mbps, UL: 10 Mbps
Peak spectral efficiency	0.33 bps/Hz	2.55 bps/Hz
Cell range	8 Km	4–12 Km
Cell configuration	Single sector	Single sector
Data transmission	Requires voice call connection	Packet switching, UDP data
Packet retransmission	No (serial data)	Reduced (UDP packets)
MIMO	No	2 × 2
Mobility	500 Km/h	500 Km/h
Handover success rate	≥ 99.5%	≥ 99.9%
Handover type	Hard	Soft (no data loss)

**Table 8:** Main specifications to address railway requirements.

Railway Requirements	Implementation
General specs.	Detailed requirements for GSM operation on Railways; ETSI TS 102 281 V2.3.0 (2013-07). Usage of the User-to-User Information Element for GSM Operation on Railways; ETSI TS 102 610 V1.3.0 (2013-01). Mobile communication system for railways (3GPP TS 22.289, Draft, Rel-15). Future Railway Mobile Communication System (3GPP TR 22.889 version 15.0.0 Rel-15). Application architecture for the Future Railway Mobile Communication System (FRMCS); Stage 2 (3GPP TS 23.790, Draft, Rel-15).
Voice	Point-to-point calls; VoLTE (GSMA IR. 92 v 10.0). Proximity-based services (ProSe); Stage 2 (3GPP TS 23.303 version 14.1.0 Rel-14). Service requirements for the Evolved Packet System (EPS) (3GPP TS 22.278 version 15.0.0 Rel-15). Architecture enhancements to support ProSe (3GPP TS 23.703 version 12.0.0 Rel-12). Security issues to support ProSe (3GPP TR 33.833 version 13.0.0 Release 13). LTE device to device proximity services; Radio aspects (3GPP TR 36.843 version 12.0.1 Rel-12). 3GPP enablers for OMA; PoC services; Stage 2 (3GPP TR 23.979 version 14.0.0 Rel-14). Emergency calls; MS emergency sessions: − IP Multimedia Subsystem (IMS) emergency sessions (3GPP TS 23.167 version 14.3.0 Rel-14). − IP based IMS Emergency calls over GPRS and EPS (3GPP TR23.869 version 9.0.0 Rel- 9). Group calls/Broadcast including emergency calls − Voice Broadcast Service (VBS); Stage 2 (3GPP TS 43.069 version 14.0.0 Rel-14). − Group Communication System Enablers for LTE (GCSE_LTE); Stage 2 (3GPP TS 23.468 version 14.0.0 Rel-14). − Mission Critical Voice Communications Requirements for Public Safety; NPSTC BBWG. − Public Safety Broadband High-Level Statement of Requirements for FirstNet Consideration, NPSTC Report Rev B. − Service aspects; Service principles (3GPP TS 22.101 version 15.0.0 Rel-15). − Architecture enhancements to support GCSE_LTE (3GPP TS 23.768 version 12.1.0 Rel-12). Evolved Multimedia Broadcast Multicast Services (eMBMS) (3GPP TS 23.246 version 14.1.0 Rel-14); MBMS; Protocols and codecs (3GPP TS 26.346 version 14.2.0 Release 14).
eMLPP	QoS concept and architecture (3GPP TS 23.107 version 14.0.0 Rel-14). Service-specific access control; Service accessibility (3GPP TS 22.011 version 15.0.0 Rel-15). E-UTRA; RRC; Protocol specification (3GPP TS 36.331 version 14.2.2 Rel-14). IMS multimedia telephony communications service and supplementary services (3GPP TS 24.173 version 14.2.0 Rel-14). AT command set for User Equipment (UE) (3GPP TS 27.007 version 14.3.0 Rel-14). Multimedia priority service (3GPP TS 22.153 version 14.4.0 Rel-14). Enhancements for Multimedia Priority Service (3GPP TR 23.854 version 11.0.0 Rel-11).
Call related	Call Forwarding supplementary services (3GPP TS 22.082 version 14.0.0 Rel-14). Call Waiting (CW) and Call Hold (HOLD) supplementary services; Stage 1 (3GPP TS 22.083 version 14.0.0 Rel-14). Call Barring (CB) supplementary services; Stage 1 (3GPP TS 22.088 version 14.0.0 Rel-14). Numbering, addressing and identification (3GPP TS 23.003 version 14.3.0 Rel-14).
LDA	LTE Positioning Protocol (LPP) (3GPP TS 36.355 version 14.1.0 Release 14) and Annex (3GPP TS 36.455 version 14.1.0 Rel-14). Functional stage-2 description of Location Services (LCS) (3GPP TS 23.271 version 14.1.0 Rel-14). Location Services (LCS); Mobile Station (MS) - Serving Mobile Location Centre (SMLC) Radio Resource LCS Protocol (RRLP) (3GPP TS 44.031 version 14.0.0 Rel-14).

**Table 9:** Time frame of LTE-R in Europe.

Phase	2008–2017	2018	2019	2020	2021	2022	2023	2024	2025	2026	2027	2028	2029	2030
System definition														
Transition strategy														
Develop new-generation terminals														
New-generation terminal trials														
New-generation terminal roll-out														
New-generation infrastructure trials														
New-generation infrastructure transition														
Transition complete														

**Table 10:** Hypotheses influencing the future railway environment (next 15+ years).

Parameter	Expected Evolution [59]
Organizational model	In Europe, the scenario will not change substantially. Regulation for all member states will come from the EU, but overall responsibility will continue to be held at a national level.
Voice requirements	It may change over time. Some stakeholders have indicated some interest in making use of voice communications which are barely used today (e.g., for communications with train crew and/or passenger announcements independently of the communications between driver and controller). Some of the voice functions of GSM-R, such as the REC, may cease to be critical voice requirements if alternative solutions are available (e.g., if the emergency call and halt to train movement is handled through data/signaling).
ETCS	It currently uses GSM circuit-switched data and it is being evolved to allow the operations over IP packet networks. ETCS operation over GSM-R GPRS is ongoing.
Signaling requirements	It will not change substantially over the next 15+ years.
Communications	The technologies in use will continue to change rapidly with a major evolution in networks, services and devices over 3–5 year cycles.
Applications	The demand for more data applications will increase. Innovative services needed to increase profits.
Radio spectrum	In key bands, spectrum for mobile use will continue to be in high demand, becoming increasingly scarce and costly to acquire.

**Table 11:** Advanced services for the IoT-connected railways.

Service	Reference	Techniques	Main Contributions
Predictive maintenance	Rabatel et al. [64]	Expert systems	Anomaly detection in complex maintenance operations. Precision is in all cases above 90% limiting both the number of false alarms and the number of undetected anomalies.
	Thaduri et al. [65]	State-of-the-art, analytics, sensor fusion and Big Data	Precise location of a heavy freight train and its main parameters.
	Firlik et al. [66]	Sensors, optimization procedures	Adjust the maintenance needs and track speed limits dynamically using embedded sensors. Experimental results of the implementation.
	Soh et al. [67]	State-of-the-art	Different strategies for preventive maintenance scheduling problem: hybrid genetic algorithms, ontology-based modeling, heuristic approaches and strategic gang scheduling.
	Nunez et al. [68]	Big Data	Maintenance decisions regarding railway tracks, all parts of the track can be monitored with appropriate intervals while maintaining the processing load within feasible limit.
	Turner et al. [69,70]	Expert systems, DSS, ontologies	Knowledge based systems to develop a prototype for maintenance scheduling.
	Canete et al. [71,72]	WSN, Zigbee	Monitoring system for slab track infrastructures using an energy consumption optimization strategy.
	Xu et al. [73]	WSN, remote monitoring	Monitor the slope deformation, the variation in the internal stress and the PPV (Peak Particle Velocity) in an existing slope adjacent to a railway track.
	Flammini et al. [74]	WSN	Early warning system for infrastructure surveillance and threat detection.
	Sa et al. [75]	Shapelet algorithms	Detecting replacement of Railway Point Machines (RPMs) using an electric current sensor.
	Ngigi et al. [76]	State-of-the-art	Applications of modern predictive control methods, analysis tools and techniques for condition monitoring systems.
	Saa et al. [77]	Ontologies, knowledge rules-based system	Tool to design complex infrastructures.
Advanced monitoring	Ostachowicz et al. [78]	State-of-the-art	Trends in SHM
	Kouroussis et al. [79]	State-of-the-art	Overview about the static and dynamic behaviour of ballasted railway tracks in SHM. Estimation of stress transfer from the train passage to the track using predictive numerical models.
	Aygün et al. [80]	State-of-the-art, WSN	General applications, SHM network topology and deployments, hardware/software properties, communication protocols and standards; and energy harvesting solutions.
	Wang et al. [81]	State-of-the-art, WSN	Integration of different types of sensors for SHM.
	Giannoulis et al. [82]	State-of-the-art, WSN	Qualitative and quantitative analysis of WSN requirements, accurate timing and synchronized sensing for high sampling rate sensors.
	Kolakowski et al. [83]	Sensors, ultrasonic probeheads, numerical models	Tests over a railway truss bridge.
	Lai et al. [84]	Sensors	Development and experimental results of a liquid level sensor based on a fiber Bragg grating for monitoring differential settlement of railway track.
	Berlin et al. [85]	WSN, feature extraction	Analysis of the vibration patterns caused by trains passing by.
	Chen et al. [86]	Sensors, optical imaging, knowledge-based systems	Monitor rail damage in the turnout zone.
	Hodge et al. [87]	State-of-the-art Sensors, WSN	Review of network design for condition monitoring.
	Chen et al. [88]	High-level programming abstraction, WSN, middleware	Practical application for SHM, results obtained using the Cooja simulator.
	Val et al. [10]	WSN	Time-synchronized network for SHM, the design includes channel measurements, network topology and architecture, physical and MAC layer design and network discovery. Performance evaluation show maximum sampling synchronization jitter values within 1 μs for sensor nodes belonging the same base station, and 2 μs for nodes of different base stations.
	Li et al. [89]	Artificial intelligence, dynamic programming and genetic algorithms	Modeling the physical topology optimization for SHM.
	Bischoff et al. [90]	WSN	Bridge structural monitoring based on events to achieve energy efficient operation.
	Franceschinis et al. [91]	WSN	Predictive monitoring of train wagon conditions. Performance, based on ns-2 simulation results, suggests that the combined use of WSN and Wi-Fi in a hierarchical architecture is adequate for long trains (e.g., several coaches) and a large number of sensing nodes.
	Anjali et al. [92]	WSN	Zigbee-based collision avoidance system that relies on vibration sensors.
Video security	Ambellouis et al. [93]	State-of-the-art	Analysis of surveillance systems, architectures, detection and analysis of complex events, onboard surveillance, applications to railway transport and review of the main worldwide projects.
	Bochetti et al. [94]	Video analytics, artificial intelligence	Security management system integrating heterogeneous intrusion detection, access control, intelligent video-surveillance and sound detection devices. Probability of detection of at least the 80% for most alarms (including motion detection, unattended luggage, yellow line crossing) and a false alarm rate of less 10 nuisance alarms per day.
	Li et al. [95]	System framework	Comprehensive video surveillance and management platform, successfully applied in the operation of Suzhou Subway Line 1.
	Flammini et al. [96]	Bayesian networks	Framework with detection models for the evaluation of threat detection.
Operations	Zhang et al. [97]	IoT, complex event processing	Design of Electric Multiple Unit (EMU) IoT-system oriented to Maintenance, Repair and Operation (MRO) including holographic train visualization and alerts.
	Briola et al. [98]	Ontology, natural language processing	Management of data collected from the centralized traffic control, improvement of the user interface through the exploitation of natural language queries.
	Tutcher et al. [99]	Ontology, natural language processing	Asset Monitoring As A Service (AMaAS).
	Fu et al. [100]	Decision support system, heuristics	Integrated hierarchical approach for creating line plans
	Yang et al. [101]	Human-computer interaction, mathematical models	System for completing cyclic train timetables in high-speed railway scenarios
	Wegele et al. [102]	Decision support systems, rescheduling algorithms	Dispatching support tools for re-ordering trains in case of delays.
	Ho et al. [103]	Particle Swarm optimization (PSO)	The performance of PSO is evaluated by comparing the service quality of the resulting timetables obtained from a sequential timetable generation approach.
	Albrecht et al. [104]	Heuristics	Space search to re-schedule timetable in case of infrastructure maintenance to minimize total delay and maximum train delay.
	Tan et al. [105]	Discrete-event optimization model	Optimization algorithm for the real-time management of a complex rail network.
PIS	Ai et al. [106]	State-of-the-art	Combination of passenger loading information from trains with social networking.
	Stelzer et al. [107]	Architecture design	Information exchange for connection dispatching, optimization of the interchange times for existing connections in intermodal transport.
	Fingar et al. [108]	Sensors, RFID, QR and NFC	Solution that enables the use of phones for acquiring electronic public transport ticket.
	Chiltern Railways [109]	Sensors, bluetooth	Application that open gates and determine the journeys taken.
FIS	Scholten et al. [110]	WSN	Monitoring integrity of cargo trains.
	Zarri et al. [111]	Business rules, knowledge representation, W3C languages	Checking rail transport of hazardous materials.
	Nan et al. [112]	WSN	Monitoring of rolling bearing in freight trains, comparison of different routing protocols and use of data compression and coding schemes based on lifting integer wavelet and Embedded Zerotree Wavelet (EZW) algorithms.
	Casola et al. [113,114]	WSN, embedded systems, cryptography	Monitoring of freight trains transporting hazardous materials. Analysis on network performance by measuring the packet loss rate on different nodes in two working conditions: train standing in the station and train running.
	Tumuler et al. [115]	Instrumentation, numerical analysis	Performance monitoring of track transitions under different loading environments. Identification of different factors contributing towards this differential movement, as well as development of design and maintenance strategies to mitigate the problem.
	Crevier et al. [116]	Operations planning, bilevel optimization	Revenue management for rail freight using bilevel mathematical formulation which encompasses pricing decisions and network planning.
	Bilegan et al. [117]	Multi-commodity flow problem, probabilistic mathematical model	Revenue management policy to dynamically accept/reject transportation requests in favor of forecasted demands with higher potential profit.
	Sirikijpanichkul et al. [118]	Agent-based modelling, ontologies	Model for evaluating decisions on the positioning of road-rail inter-modal freight hubs.
	Luo et al. [119]	Dynamic forecasting, stochastic comparison	Revenue management in intermodal transportation.
	Wang et al. [120]	Stochastic resource allocation	Resource management for containerized cargo transportation.
	Masoud et al. [121]	Mixed integer programming, heuristics	Scheduling optimization of the performance of sugarcane rail transport system.
Autonomous systems, safety assurance and signaling systems	Dominguez et al. [122]	ATO speed profile	A computer aided procedure for the design of optimal speed profiles for automatic subway and light rail systems. The newly designed profiles result in 20% of savings versus the one already in use. Taking into account the implementation of an on board storage device, up to 47.5% of savings could be expected.
	Guo et al. [123]	ATP driver-machine interface, GUI model	Interface for controlling over-speeding automatically.
	Salmane et al. [124]	Dempster–Shafer, hidden Markov model	Detecting hazard situations at level crossings with video analytics.
	Govoni et al. [125]	State-of-the-art, fixed object scanner algorithm	Surveillance of railway crossing areas with UWB.
	Goverde and Meng [126]	Data collection and processing	Detection of conflicts due to timetable flaws or capacity bottlenecks.
	Kecman et al. [127]	Timed-event graph model, prediction algorithm	Model for predicting accurately the timing of certain train events.
	Kecman et al. [128]	Process mining	Automatic identification of route conflicts with conflicting trains, arrival and departure times/delays at stations, and train paths on track section and blocking time level.
	Corman et al. [129]	Advanced mathematical models, automatic tools for rescheduling traffic in real-time	Real-time control of railway traffic.
	Sama et al. [130]	Alternative graph, disjunctive programming, metaheuristic algorithms	Fast scheduling and routing trains in complex and busy railway networks.
	Marais et al. [21]	State-of-the-art	GNSS-based solutions for signaling applications.
	Lu et al. [132]	Stochastic Petri net model	GNSS and sensor fusion in train localization.
	Aboelela et al. [133]	WSN, fuzzy data aggregation	Multi-layered and multi-path routing architecture to predict inclinations in track.
	Daliri et al. [134]	WSN, fuzzy logic, sensors	Image processing and electromagnetic detection of hazardous objects.
	Wang et al. [135]	WSN	Monitoring system for early earthquake detection.
	Wu et al. [140]	Key management protocols, cryptography	Secure train-to-train communication schemes: autonomous train-to-train channel with asymmetric cryptographic primitives and quasi-autonomous train-to-train channel with symmetric cryptographic primitives.
	Chan et al. [141]	Key update scheme	Secure key establishment for train-to-infrastructure networking.
	Bennetts et al. [142]	State-of-the-art	Securing railways: plans against the identified threats.
	Greenberg et al. [143]	Simulation tools	Models that replicate rail passenger traffic flows, model to trace chemical plumes released by a slow-moving freight train, model that estimates the regional economic consequences of a variety of rail-related hazard events.
Energy efficiency	Xun et al. [136]	Analytical methods of coordinated train control	Fully automatic operation system by modifying the running time between adjacent stations.
	Gruden et al. [137]	WSN, remote sensing, energy scavenging	Monitoring the wheel bearings, the number of successfully transmitted messages per day is in average about 92%, lost messages are caused by fading dips or mechanical damages of the sensors.
	Hamid et al. [138]	Genetic algorithms	Design of an optimized train trajectory, energy by up to around 25% can be saved.
	Bocharnikov et al. [139]	Genetic algorithms	Optimal train trajectories in electrically powered suburban railways. Energy savings of up to 40% may be achieved for a 10% increase in journey time.

## Abbreviations

The following abbreviations are used in this manuscript:

3GPP	3rd Generation Partnership Project
ASCI	Advanced Speech Call Items
BS	Base Station
BSC	Base Station Controller
CA	Carrier Aggregation
CBTC	Communications Based Train Control
CCBG	Critical Communication Broadband Group
CCTV	Closed-Circuit Television
CoMP	Coordinated Multi-point
DL	Downlink
DSS	Decision Support System
EGPRS	Enhanced General Packet Radio Service
EIRENE	European Integrated Railway Radio Enhanced NEtwork
eLDA	enhanced Location Dependent Addressing
eMBMS	Evolved Multimedia Broadcast Multicast Service
eMLPP	enhanced Multi-Level Precedence and Pre-emption
EMU	Electric Multiple Unit
eREC	enhanced Railway Emergency Call
ERA	European Railway Agency
ERTMS	European Rail Traffic Management System
ETCS	European Train Control System
ETSI	European Telecommunications Standards Institute
FA	Functional Addressing
FRS	Functional Requirements Specification
GCR	Group Call Register
GNSS	Global Navigation Satellite Systems
GSMA	GSM Association
GSM-R	Global System for Mobile Communications-Railways
HMI	Human-Machine Interface
IMS	IP Multimedia Subsystem
IMT-Advanced	International Mobile Telecommunications - Advanced
IoT	Internet of Things
QoE	Quality of Experience
QoS	Quality of Service
LAS	Link Assurance Signal
LDA	Location Dependant Addressing
LTE-A	LTE-Advanced
M2M	Machine-to-Machine
MAC	Medium Access Control
MBMS	Multimedia Broadcast Multicast Service
MBSFN	Multicast and Broadcast over Single Frequency Networks
MCPTT	Mission Critical Push To Talk over LTE
MRO	Maintenance, Repair and Operation
MS	Mobile Station
OFDM	Orthogonal Frequency Division Multiplexing
PoC	Push-to-Talk over Cellular
ProSe	Proximity Services
RAMS	Reliability, Availability, Maintainability and Safety
SIL	Safety Integrity Level
TCC	Train Control Center
TEDS	TETRA Enhanced Data Service
TETRA	Trans European Trunked RAdio
UIC	Union Internationale des Chemins de Fer
UL	Uplink
VBS	Voice Broadcast Service
VGCS	Voice Group Call Service
VoLTE	Voice over LTE
WiMAX	Worldwide Interoperability for Microwave Access
WLAN	Wireless Local Area Network
WSN	Wireless Sensor Networks
